# Decision volume and temporal workload structure as dissociable correlates of fatigue and mood in intensive care physicians: a real-time observational study

**DOI:** 10.1186/s13054-026-06249-6

**Published:** 2026-10-17

**Authors:** Felix Hieber, Lorenz Schiessl, Anne Herrmann, Cynthia Kohl, Moritz Lux, Sebastian Blecha, Dirk Lunz, Martin Kieninger, Christoph Eissnert, Martin G. Kees, Alexander L. Leibold

**Affiliations:** 1 Department of Anaesthesiology and Intensive Care Medicine, Franz-Josef-Strauss-Allee 11, Regensburg, D-93051 Germany; 2 University Department of Obstetrics and Gynaecology, Clinic St. Hedwig of The Order of St. John, University of Regensburg, Steinmetzstr. 1-3, D-93049 Regensburg, Germany; 3 Department of Epidemiology and Preventive Medicine, Medical Sociology, University Hospital of Regensburg, Franz-Josef-Strauss-Alle 11, D-93053 Regensburg, Germany; 4 Department of Anaesthesiology and Intensive Care Medicine, University Hospital of Regensburg, Franz-Josef-Strauss-Allee 11, Regensburg, 93053 Germany

**Keywords:** Decision fatigue, Intensive care, Temporal workload, Physician wellbeing, Patient safety, Psychometric assessment, Mixed-effects models, Observational study

## Abstract

**Background:**

Intensive care physicians make hundreds of decisions per shift under sustained cognitive and emotional demands. Decision fatigue, the tendency towards less effortful choices as cumulative decision load increases, may affect clinician wellbeing and decision processes. However, the associations of decision volume and temporal workload structure with within-shift fatigue and mood in intensive care unit (ICU) physicians remain unclear. This study explored whether decision volume and temporal clustering show distinct associations with fatigue, sleepiness, mood, and post-shift decision responses.

**Methods:**

Five physicians (two residents, three consultants) at a surgical ICU of a German university hospital were shadowed during 38 twelve-hour shifts (456 hours) between October and December 2025. Clinical decisions were classified by severity using a study-specific, non-validated Decision Severity Score. Because recruitment was feasibility-based and involved only five physicians, all analyses were considered exploratory and hypothesis-generating. Psychometric assessments of decision fatigue, sleepiness, and mood (Decision Fatigue Scale, Karolinska Sleepiness Scale, Profile of Mood States – Short Form) were administered at morning, midday, and shift end. Following each shift, physicians completed a standardised scenario task requiring three binary treatment decisions. Temporal workload structure was quantified using the burstiness metric; shifts were classified into four types by median split of decision density and burstiness (quiet-smooth, quiet-clustered, busy-smooth, busy-clustered). Linear and binomial mixed-effects models with physician as random intercept were used throughout.

**Results:**

Across 17,953 recorded decisions (median 474 per shift; range 366–589), a higher mean decision rate was associated with greater end-of-shift decision fatigue: an increase of 0.5 decisions per 5-minute interval corresponded to a 2.1-point higher decision-fatigue score (95 % CI 0.6 to 3.6). Higher burstiness was associated with less mood disturbance, with a 0.10 increase in burstiness corresponding to a 6.3-point lower mood score (95% CI −10.2 to −2.3). Averaged across all shifts, changes between morning and evening were small and not statistically significant for any of the three scores. Within-shift trajectories differed by shift type, however, with mood disturbance increasing over the shift in quiet-smooth shifts only, a difference already apparent at midday. Quiet-smooth shifts, the least objectively demanding, showed the least favourable psychometric values. Response certainty in the post-shift scenario task was lower after shifts with more task interruptions, with each ten additional interruptions corresponding to a 0.29-point lower certainty score (p = 0.012), whilst a descriptive trend towards more conservative scenario responses was observed in busy-clustered shifts (odds ratio 2.25, p = 0.555).

**Conclusions:**

Decision volume and temporal workload structure showed different associations with psychometric outcomes: higher decision rates were associated with greater end-of-shift decision fatigue, whereas greater temporal clustering was associated with lower mood disturbance, already apparent from midday onwards. That more quiet, uniformly paced shifts showed the least favourable psychometric values suggests that reducing decision volume alone may not improve physician wellbeing; this observation requires replication. Mitigation strategies may therefore need to extend beyond reducing decision volume to protecting concentrated, meaningful clinical activity and minimising hindrance stressors such as interruptions. Future multicentre studies should determine whether these associations are reproducible and whether temporal workload structure is related to objective decision quality or patient-safety outcomes.

**Trial registration:**

German Clinical Trials Register, DRKS00037564. Registered 27th August 2025.

**Supplementary Information:**

The online version contains supplementary material available at https://doi.org/10.1186/s13054-026-06249-6.

## Background and research question

Decision-making is a core competence of intensive care medicine. Intensivists make large numbers of complex, high-stakes decisions during each working day, ranging from minor ventilator adjustments and sedation titration to more or less far-reaching crossroads such as anti-infective treatment choices, advanced diagnostic or therapeutic procedures, and end-of-life decisions. Many of these directly affect survival, long-term outcomes, and resource use in critically ill patients. Even in settings with detailed guidelines and decision-support tools, the appropriateness and timing of diagnostic and therapeutic choices ultimately depend on the individual clinician, who must integrate incomplete information under time pressure, resource constraints, and emotional strain. Under such conditions, cognitive shortcuts and biases are more likely to occur and may compromise decision quality and patient safety [1].

Beyond these situational factors, cumulative decision load itself has been proposed as a key determinant of clinical decision-making. In a recent systematic review, Maier et al. defined decision fatigue as “the tendency towards making less effortful decisions as the cumulative mental burden of effortful decision-making increases” [2]. This definition emphasises a qualitative shift in decision strategies rather than a simple increase in errors. This also aligns with broader theoretical accounts of cognitive depletion, including the ego depletion model [3], the self-regulatory model of resource scarcity [4], and the opportunity cost model of mental effort [5]. Across quantitative studies in healthcare professionals, Maier et al. found that 45% reported statistically significant decision fatigue effects on medical decision-making, such as a greater use of status-quo or default options later in a shift [2]; the remaining 55%, however, found no significant effects, underscoring the heterogeneity of the evidence base. A complementary systematic and scoping review likewise concluded that clinical decision fatigue is a plausible and clinically relevant construct, but highlighted considerable gaps in conceptual clarity and measurement [6]. Perry et al. further noted that only approximately one quarter of decision fatigue studies in healthcare evaluate decisions against predefined quality standards, with the remainder relying primarily on proxy outcomes such as choice patterns or self-reported fatigue [7]. As a result, it remains unclear to what extent observed changes in decision behaviour reflect meaningful deterioration in clinical decision quality.

Importantly, the burden associated with clinical decision-making does not appear to arise from deciding per se, but from the volume and perceived difficulty of the decisions to be made. At the same time, engaging with complex, meaningful clinical problems is a source of professional fulfilment: clinicians frequently report burnout and moral distress alongside substantial professional satisfaction [8–10]. Understanding decision fatigue in this setting therefore requires a perspective that captures both the detrimental effects of cumulative decision load and the potentially energising character of demanding clinical work.

A dimension largely overlooked in decision fatigue research is the temporal structure of workload. Most existing studies treat decision load as a cumulative quantity, without considering how decisions are distributed in time. Yet the same number of decisions may be experienced very differently depending on whether they arrive at a steady rate or in dense, irregular bursts – a distinction that can be quantified using the burstiness metric [11]. Whether temporal workload structure predicts psychometric and behavioural outcomes in ICU physicians has not previously been examined.

Given the feasibility-based sample and the absence of a confirmatory sample-size calculation, the study was designed as an exploratory investigation, and all inferential analyses should be interpreted as hypothesis-generating rather than confirmatory.

Against this background, we addressed the following research questions:

1. How many clinical decisions do ICU physicians make during a twelve-hour shift, and how can they be categorised by type and perceived severity?
2. How are decision volume and temporal workload structure associated with within-shift changes in fatigue-related psychometric scores?
3. Can workload characteristics be used to derive meaningful shift-type profiles with distinct psychometric trajectories?

As an additional, strictly exploratory objective, we examined to what extent workload characteristics and within-shift fatigue changes are reflected in standardised scenario decision-making, particularly regarding conservative versus active treatment choices [2, 7].

## Methods

The study employed a real-time observational design in which ICU physicians were shadowed during routine clinical shifts, combining continuous decision monitoring with repeated psychometric assessments and a standardised experimental task to capture both the objective workload structure and the subjective experience of decision-making in a naturalistic clinical setting.

### Participants and recruitment

Participants were recruited among intensivists working on one of three surgical intensive care units within the Department of Anaesthesiology of a German University Hospital; all observations were conducted on the general surgical ICU. The sample comprised three consultants and three residents, reflecting different levels of clinical experience (see Table 2). One resident dropped out and was excluded from all analyses after four observed shifts due to an unplanned absence. No financial or material incentives were provided. No confirmatory sample-size calculation was performed; the number of participants and shifts was determined by feasibility and by the intensive resources required for continuous real-time observation. All participants provided written informed consent. The study was approved by the Ethics Committee of the University of Regensburg (24–3873-104) and prospectively registered in the German Clinical Trials Register (DRKS00037564) on 27 August 2025, before the start of data collection. Data were collected between October and December 2025. All procedures were conducted in accordance with the Declaration of Helsinki. Shadowing was carried out by a senior medical student (F.H.) with prior professional experience as a nurse in the ICU, who had no supervisory or hierarchical relationship with any of the participating physicians. He was trained in standardised data collection procedures by the research team prior to observations and took no part in patient care.

### Pilot phase

A one-week pilot phase was conducted in February 2025 to finalise study procedures, including the shadowing protocol, the real-time data collection method, and the calibration of the Decision Severity Score. Two physicians who subsequently participated in the main study (a02 and a05) were each shadowed for one shift during this phase; two additional residents were also observed but left the unit owing to planned staffing rotations prior to main data collection. Data from the pilot phase were not included in any analysis.

### Decision recording and severity classification (Research question 1)

Participants were shadowed during twelve-hour daytime shifts (07:00–19:00), yielding 38 observed shifts. Medical decisions, interruptions, and breaks were recorded using a digital tally counter application (Counter Tally Count, version 2025.4.1; Tevfik Yucek, Mikrasya, iOS).

Interruptions were defined as externally triggered, unplanned disruptions to an ongoing task requiring immediate attentional reorientation, such as a nurse’s question during handover or an unexpected phone call mid-ward round [12].

Rest breaks were defined as planned, self-initiated periods of work cessation within a shift, intended to facilitate recovery from accumulated fatigue [13]. Breaks included both the legally mandated work interruptions (45 min per 12-hour shift, typically divided into a coffee and a lunch break) and any additional brief pauses that occurred at the physician’s discretion depending on workload. Importantly, physicians remained on the ICU throughout all breaks, continued to respond to phone calls, and remained available to nursing staff for any queries; no physician was unreachable or off-site during any recorded break. Observation days were deliberately scheduled on days without formal teaching sessions, departmental meetings, or comparable off-unit commitments, so that no physician left the unit at any point during an observed shift.

Clinical decisions were classified using a study-specific Decision Severity Score developed *de novo*, as no validated instrument was available. Drawing on a preceding qualitative study by our group [10], the score comprises six dimensions rated on three-point scales (sum score 6–18; small: 6–8; medium: 9–14; large: 15–18; see Table 1). Prototypic examples include discontinuing a stable medication (*small*), extubating a patient after a successful breathing trial (*medium*), and initiating ECMO in refractory septic shock (*large*).

**Table 1:** Decision Severity Score

Dimension	Criterion	Score
Risk to the patient	Low	1
	Moderate but controllable	2
	High, life-threatening	3
Complexity of the decision	Simple, standard procedure	1
	Complex, requires experience	2
	Highly complex, multiple variables	3
Consequences	Short-term, minor	1
	Reversible, non-critical	2
	Potentially irreversible	3
Time pressure	Within 12 h	1
	Moderate urgency, within 6 h	2
	Acute, emergency	3
Evidence base	Strong evidence, SOP available	1
	Inconclusive evidence	2
	Unclear or absent	3
Physician experience	$$$$$$$$$$$$$$$$$${>}5$$$$$$$$$$$$$$$$$$years ICU experience	1
	2–5 years ICU experience	2
	0–2 years ICU experience	3
Sum score 6–8		Small
Sum score 9–14		Medium
Sum score 15–18		Large

Each of the six dimensions is rated on a three-point scale; the sum score ranges from 6 to 18

Decisions were classified as small (S: 6–8), medium (M: 9–14), or large (L: 15–18). SOP = standard operating procedure

### Psychometric assessments (Research question 2)

Three established psychometric instruments were self-administered by the participating physicians at the start of the shift, at midday, and at shift end. The Decision Fatigue Scale (hereafter: decision-fatigue score) comprises ten statements rated on a five-point agreement scale (total score 10 to 50) and quantifies decision-related exhaustion; it was originally evaluated in surrogate decision makers of critically ill patients [14], and higher scores indicate greater fatigue. The Karolinska Sleepiness Scale (hereafter: sleepiness score) is a single-item measure with nine verbally anchored response options, ranging from extremely alert to extremely sleepy, which has been shown to be sensitive to insufficient sleep and impaired waking function [15]; higher values indicate greater sleepiness. Mood was assessed with the Profile of Mood States – Short Form, a 37-item version of the original 65-item instrument [16]. Items are rated from 0 (not at all) to 4 (extremely) and form six subscales: tension-anxiety, depression-dejection, anger-hostility, vigour-activity, fatigue-inertia, and confusion-bewilderment. The total mood disturbance score (hereafter: mood score) is calculated as the sum of the five negative subscales minus the vigour subscale, giving a possible range of *-24* to *+124*, with higher values indicating greater mood disturbance. Its six-factor structure and internal consistency (Cronbach’s *α = 0.78*to 0.91) have been confirmed in independent samples [17].

### Workload metrics and shift-type classification (Research questions 2 and 3)

Workload metrics comprised mean decision rate per 5-minute interval, burstiness of decision timing, number of interruptions, and break characteristics. All metrics were computed across a full 144-bin observation window (12 h *×* 5 min). A decision severity index was calculated by assigning ordinal weights to each decision category (small = 1, medium = 2, large = 3) and computing their mean across all decisions in a shift, thereby quantifying the average clinical complexity of the shift’s decision load. The decision severity index thus summarises, at shift level, the per-decision severity classification described above.

Burstiness was defined as $$$$$$$$$$$$$$$$$$B = (\sigma - \mu )/(\sigma + \mu )$$$$$$$$$$$$$$$$$$, where *μ* and *σ* denote the mean and standard deviation of decisions per bin [11]. *B* ranges from *-1* (perfectly regular) to *+1* (maximally clustered). As the metric was originally developed for inter-event intervals, its application to decision counts within fixed time bins represents a methodological adaptation.

Shifts were classified along two dimensions, decision density (mean rate) and temporal clustering (burstiness), dichotomised at the sample median, yielding four types: *quiet-smooth*, *quiet-clustered*, *busy-smooth*, and *busy-clustered*.

### Post-shift scenario task (Exploratory objective)

Following each shift, participants completed a standardised clinical scenario requiring three consecutive binary decisions, rating their confidence in the chosen response on a five-point Likert scale (1 = not at all confident; 5 = completely confident). Eight scenarios were used across the observation period (one per shift), developed by three senior ICU consultants (M.K./S.B./D.L.) not otherwise involved in the study. Each decision corresponded to category 5 (therapeutic procedure-related) of the clinical decision taxonomy by Ofstad et al. [18] and framed as a choice between a conservative decision and an active decision [19, 20]. Scenarios ranged from 598 to 994 words in length and incorporated laboratory findings and radiological results.

### Statistical analysis

Each physician contributed several shifts, so observations from the same physician are related. All models therefore included a random intercept for physician, which separates stable differences between individuals from shift-to-shift variation; the intraclass correlation coefficient (ICC) describes how much of the total variability these stable differences explain.

Research question 1 (decision volume and severity) required no statistical testing: decision counts, severity categories, and workload metrics were summarised descriptively.

For research question 2 (workload and psychometric change), we tested whether the five workload metrics (mean decision rate, burstiness, interruptions, and the number and duration of breaks) predicted the evening decision-fatigue, sleepiness, and mood scores, using linear mixed-effects models with all five predictors entered simultaneously.

For research question 3 (shift-type profiles), we compared the four shift types on the same evening scores using linear mixed-effects models with shift type as a fixed factor, first unadjusted and then adjusted for total decision count, decision severity index, interruptions, and breaks. To examine whether differences between shift types emerged early in the shift or only towards its end, additional linear mixed-effects models used all three timepoints per shift, with random intercepts and time slopes for physicians and for shifts within physicians.

For the exploratory scenario objective, we tested whether workload or shift type predicted conservative scenario responses, using binomial (logistic) mixed-effects models with odds ratios derived via estimated marginal means [21], and response certainty, using linear mixed-effects models. Physician-level patterns (*n = 5*) were described without statistical testing. As a sensitivity check, the workload models were repeated with each score averaged across the three daily measurements instead of the evening value.

Model estimates (*b*) give the change in an outcome per one-unit change in a predictor; odds ratios (ORs) are reported with 95 % confidence intervals; *p*-values for fixed effects were obtained with Satterthwaite’s approximation, and two-tailed *p < 0.05* defined statistical significance. Analyses were performed in R [22] with lme4 and lmerTest [23, 24] and reviewed by a professional biometrician at the University Hospital of Regensburg (see Acknowledgements). Distributions of all outcomes, residual diagnostics, and robustness analyses are reported in the Supplements S4 and S8.

## Results

### Participants and observed shifts

Five ICU physicians (two residents and three consultants, see Table 2) participated following the exclusion of one resident (a04). Four physicians completed eight twelve-hour shifts each and one was observed for six shifts, yielding 38 shifts and 456 hours of observed ICU work. Across all shifts, 17,953 clinical decisions were documented: 16,107 (89.7%) small, 1,831 (10.2%) medium, 8 (0.04%) large, and 7 (0.04%) end-of-life decisions; the latter were retained in the total count but not analysed separately (shift-level details are provided in Supplementary Table S15).

**Table 2:** Characteristics and psychometric profiles of participating physicians

Physician	Role	ICU exp. (yr)	Work exp. (yr)	Shifts	Fatigue		Sleepiness		Mood	
				(*n*)	*M*	*Δ M*	*M*	*Δ M*	*M*	*Δ M*
a01	Res.	1–2	1–3	8	18.9	+1.6	3.1	−0.2	−8.6	−0.9
a02	Res.	1–2	1–3	6	11.7	+1.3	5.5	+1.3	−0.7	+7.5
a03	Cons.	7–10	10–15	8	14.5	+2.5	3.0	+0.8	−3.6	+4.6
a04	Res.	excluded		—	—					
a05	Cons.	$$$$$$$$$$$$$$$$$${>}10$$$$$$$$$$$$$$$$$$	$$$$$$$$$$$$$$$$$${>}20$$$$$$$$$$$$$$$$$$	8	10.8	−2.5	2.1	−1.0	−12.1	−9.9
a06	Cons.	1–2	10–15	8	30.6	+0.9	5.4	+0.6	+28.8	−1.8

ICU and work experience are given as ranges; at the explicit request of the participating physicians, more precise values are not reported, to preserve anonymity. *M* = mean evening score across all observed shifts;*Δ M*= mean change from morning to evening (positive values indicate deterioration). Physician a04 was excluded from all analyses owing to an unplanned absence. Res. = Resident; Cons. = Consultant. Fatigue, Sleepiness, and Mood denote the evening decision-fatigue, sleepiness, and mood scores (see Methods)

### Decision volume, severity, and temporal structure (Research question 1)

Physicians made on average 472 decisions per shift (*SD = 53*; range: 366–589), at a mean rate of 3.27 per 5-minute interval (*SD = 0.36*). The decision severity index averaged 1.10 (*SD = 0.04*), corresponding to approximately 10% of decisions being of medium or greater complexity. Burstiness averaged *B = 0.38* (*SD = 0.06*; range: 0.29–0.55); all shifts showed positive values, indicating that temporally clustered decision patterns were the norm. Decision density was consistently highest during the first half hour of the shift (Fig. 2), reflecting the structured morning handover, during which every patient is reviewed systematically, including medication, haemodynamics, and a full ABCDE assessment.

### Workload metrics and psychometric outcomes (Research question 2)

**Fig. 1:**
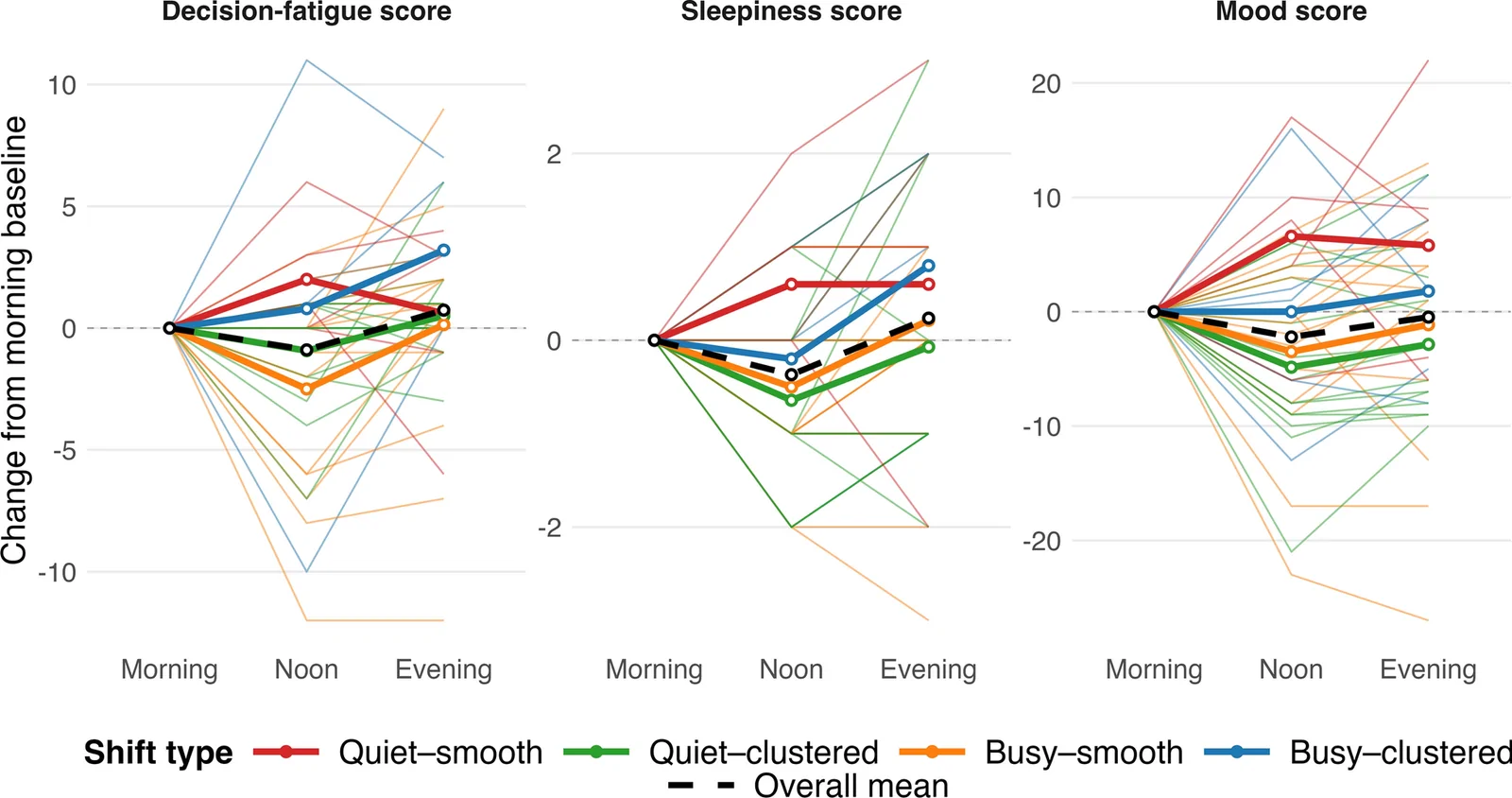
Within-shift trajectories of the decision-fatigue, sleepiness, and mood scores across 38 observed shifts. Values are absolute changes from the morning baseline. For all three scores, higher values indicate less favourable states. Thin lines represent individual shifts and thick coloured lines the mean per shift type. Coloured lines show unadjusted group means. The thick dashed black line represents the overall mean across all shifts

Evening scores varied considerably between shifts: the decision-fatigue score averaged 17.6 (*SD = 7.9*; range 10–34), the sleepiness score 3.7 (*SD = 1.7*; range 2–7), and the mood score 0.8 (*SD = 16.1*; range *-15* to 44). In mixed-effects models using all three timepoints per shift, mean changes from morning to evening were small and showed no clear evidence of deterioration for any of the three scores (decision-fatigue score: *b = +0.74*, 95 % CI *-0.71* to 2.18; sleepiness score: *b = +0.28*, 95 % CI *-0.90* to 1.45; mood score: *b = -0.17*, 95 % CI *-8.69* to 8.34). Within-shift trajectories differed by shift type, however: in quiet-smooth shifts, mood disturbance increased from morning to noon (*b = +7.9*, *SE = 3.3*, *p = 0.020*) and remained elevated in the evening (*b = +8.5*, *SE = 4.2*, *p = 0.059*), whereas all other shift types differed significantly from this trajectory (interaction terms at noon: all *p ≤ 0.036*; Fig. 1, Supplementary Section S7).

In mixed-effects models, a higher mean decision rate was associated with a higher evening decision-fatigue score (*b = 4.13*, *SE = 1.50*, 95 % CI 1.18 to 7.14, *p = 0.010*; Table 3). Burstiness was associated with a lower evening mood score (*b = -62.6*, *SE = 20.3*, 95 % CI *-102.0* to *-23.0*, *p = 0.004*), with a similar tendency for the sleepiness score (*b = -7.57*, *SE = 3.77*, *p = 0.055*). Interruptions, breaks, and break duration showed no clear associations with the psychometric outcomes; the corresponding confidence intervals were wide, and clinically relevant associations cannot be excluded.

Intraclass correlation coefficients were high for all three primary psychometric outcomes (decision-fatigue score: ICC = 0.92; sleepiness score: ICC = 0.69; mood score: ICC = 0.89). A substantial proportion of the variability in the outcomes was therefore attributable to stable differences between physicians rather than to variation between shifts.

### Shift-type profiles (Research question 3)

**Fig. 2:**
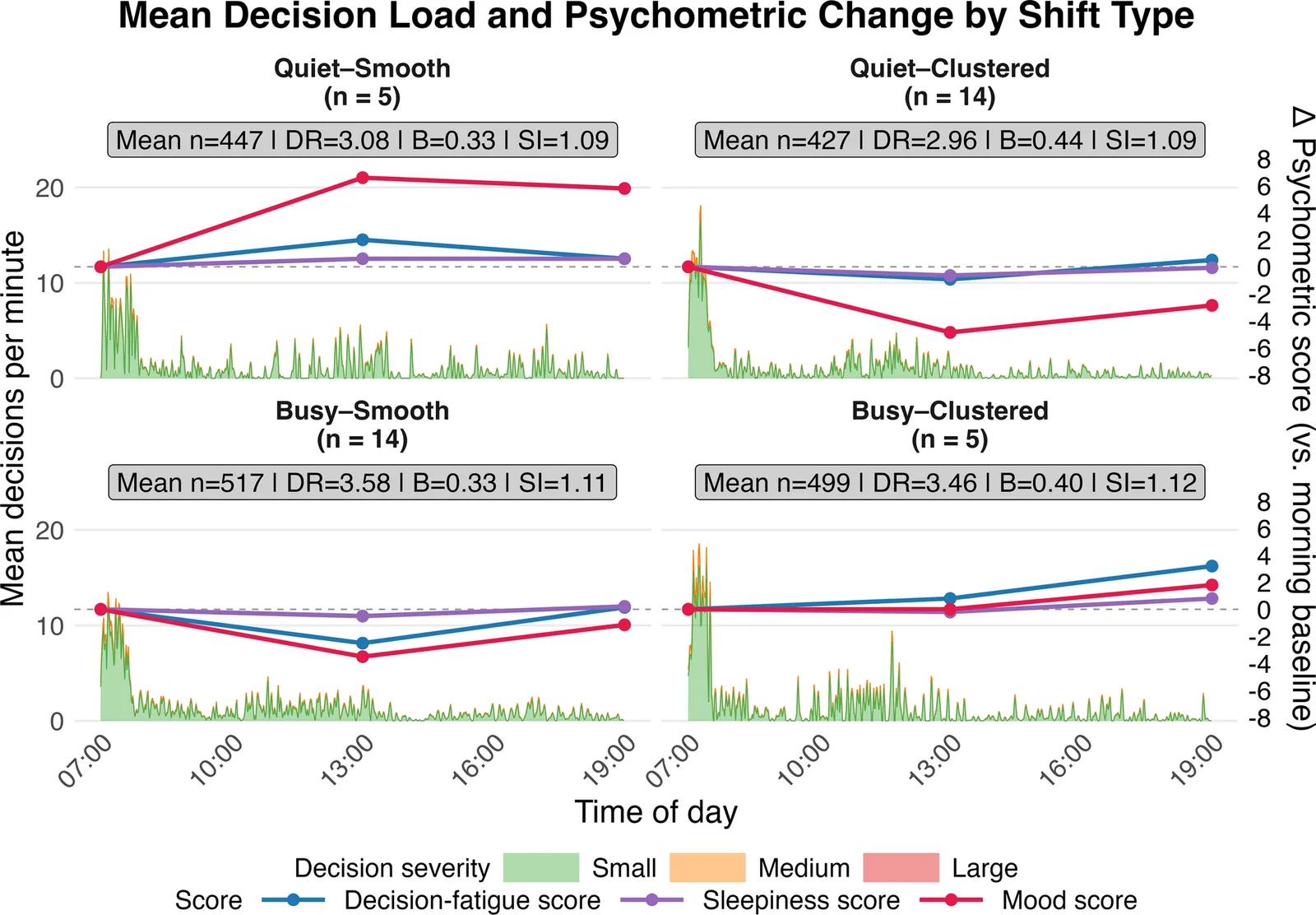
Mean decision load and psychometric change by shift type. Shaded areas (left axis) show the mean number of decisions per minute across all shifts of each type, stacked by decision severity (green = small, orange = medium, red = large). Lines (right axis) show the mean change in the decision-fatigue, sleepiness, and mood scores from the morning baseline; the dashed horizontal line indicates no change. Annotation boxes give shift-type means: *n* = total decisions per shift, DR = mean decision rate per 5-minute interval, B = burstiness, SI = decision severity index

Of the 38 shifts, five were classified as quiet-smooth, 14 as quiet-clustered, 14 as busy-smooth, and five as busy-clustered. Descriptively, quiet-smooth shifts showed the least favourable evening values on all three scores (decision-fatigue score *M = 22.2*; sleepiness score *M = 4.8*; mood score *M = 22.6*; Supplementary Section S5), although this group comprised only five shifts; mean decision load and score trajectories by shift type are shown in Fig. 2.

In mixed-effects models, shift type was associated with the mood score, both unadjusted and adjusted for total decision count, decision severity index, interruptions, breaks, and break duration: quiet-smooth shifts showed substantially higher mood disturbance than all other shift types (all *p ≤ 0.002* in adjusted models). Shift type was not significantly associated with the decision-fatigue or sleepiness score. Among the adjustment covariates, a higher decision severity index was independently associated with a lower mood score (*b = -76.1*, *SE = 26.3*, *p = 0.007*) and a lower sleepiness score (*b = -11.9*, *SE = 5.84*, *p = 0.048*), whilst a higher total decision count was associated with a higher decision-fatigue score (*b = +0.03*, *SE = 0.01*, *p = 0.046*). Full model estimates are provided in Supplementary Table S6.

In the mixed-effects models using all three timepoints per shift, mood-score differences between quiet-smooth and the other shift types were already apparent at midday (quiet-clustered: *b = -12.0*, *p = 0.001*; busy-smooth: *b = -11.9*, *p = 0.001*).

### Exploratory analyses: scenario decision-making and physician-level observations

**Fig. 3:**
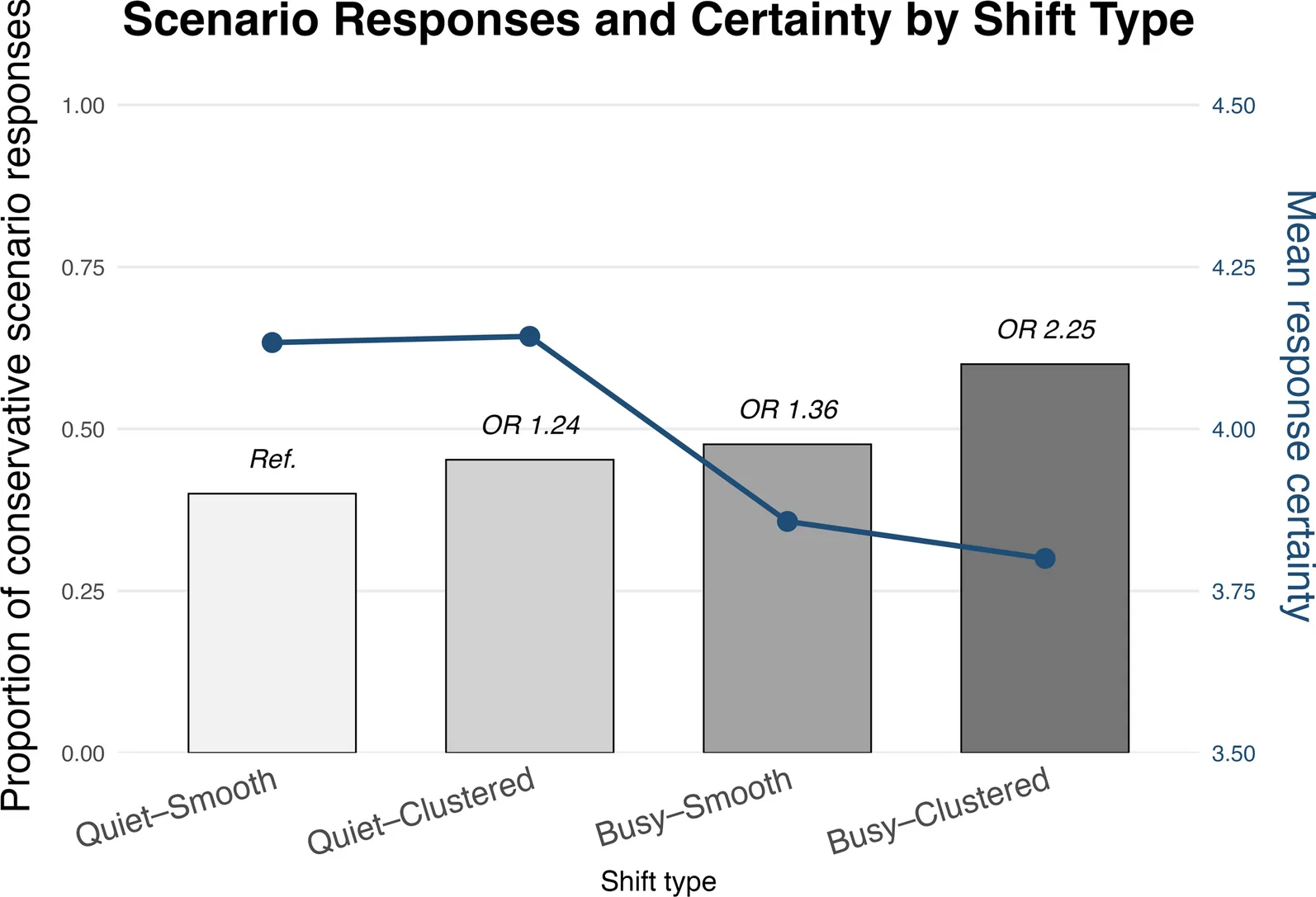
Mean proportion of conservative scenario responses (bars, left axis) and mean response certainty (line, right axis) by shift type. OR annotations are unadjusted odds ratios relative to quiet-smooth (reference); all comparisons were non-significant (*p> 0.25*), and findings should be interpreted as descriptive only given the small number of scenarios per shift (*k = 3*)

No clear association was detected between workload variables or shift type and conservative scenario responses. Descriptively, the OR for conservative responses in busy-clustered versus quiet-smooth shifts was 2.25 (95 % CI [0.52, 9.70]; adjusted OR 4.31 [0.72, 25.94]); given the small number of scenario decisions per shift (*k = 3*), these findings are strictly exploratory (Supplementary Table S7, Fig. 3). Response certainty did not differ between shift types but was lower after shifts with more task interruptions: each ten additional interruptions corresponded to a 0.29-point lower certainty score (*b = -0.029* per interruption, *SE = 0.011*, *p = 0.012*). Conservative and active responses were not interpreted as correct or incorrect decisions, as the scenarios did not define an objective quality criterion.

At the physician level (*n = 5*), mean evening decision-fatigue scores ranged from 10.8 to 30.6 and mood scores from *-12.1* to 28.8 (Table 2). The most experienced consultant showed the lowest mean evening decision-fatigue score (*M = 10.8*), the only negative mean morning-to-evening change (*M = -2.5*), and the most favourable mood trajectory (mean within-shift mood change *M = -9.9*), whilst the least experienced consultant showed the highest evening decision-fatigue score (*M = 30.6*) and the most adverse mood profile (mood score *M = +28.8*). This pattern is consistent with clinical experience as a potential moderator of susceptibility to decision fatigue. As physician identity and experience level are perfectly confounded at *n = 3* consultants, these observations cannot be disentangled from between-person differences in shift allocation or baseline mood, and are strictly hypothesis-generating.

**Table 3:** Associations between shift-level workload metrics and evening psychometric scores

Predictor	Fatigue (evening)		Sleepiness (evening)		Mood (evening)	
	*b*	*p*	*b*	*p*	*b*	*p*
Mean decision rate	**+4.13**	**0.010**	*-0.09*	0.880	*-4.86*	0.134
Burstiness	*-2.80*	0.773	*-7.57*	0.055	*-62.61*	**0.004**
Interruptions	*-0.11*	0.112	*-0.05*	0.070	*+0.04*	0.801
Breaks (count)	*+0.39*	0.621	*+0.07*	0.823	*+0.83*	0.616
Break duration (min)	*+0.02*	0.397	*-0.01*	0.520	*+0.01*	0.861

Fatigue, Sleepiness, and Mood denote the evening decision-fatigue, sleepiness, and mood scores (see Methods). Fixed-effect estimates (*b*) and *p*-values from linear mixed-effects models with physician as a random intercept. Bold =*p < 0.05*

Sleepiness score, burstiness:*p = 0.055*; interruptions:*p = 0.070*(trends)

## Discussion

Decision load showed different associations with fatigue and mood: a higher decision rate was associated with greater end-of-shift fatigue, whereas greater temporal clustering was associated with lower mood disturbance, with quiet-smooth shifts, the objectively least demanding, showing the least favourable values. In the exploratory post-shift scenarios, more task interruptions during the preceding shift were associated with lower response certainty, whilst conservative-response patterns remained descriptive.

Mean psychometric change across the shift was small, whereas higher decision rates were associated with greater evening decision fatigue, consistent with decision fatigue being a relevant phenomenon in intensive care medicine. This pattern is compatible with the strength model of cognitive depletion, which conceptualises self-control as a limited resource that is gradually consumed, so that after many effortful choices later decisions default to the less demanding option [3], although a large preregistered replication has contested this account [25]. Alternative models are the self-regulatory model of resource scarcity, in which the subjective experience of insufficient resources narrows attention and shifts priorities towards immediate goals [4], and the opportunity cost model of mental effort, in which the feeling of fatigue signals that staying on the current task brings diminishing returns compared with attending to something else [5]. It also parallels empirical findings by Allan et al., who observed increasingly conservative telephone triage decisions with time since the last rest break [26], and by Kruer et al., who found decision-fatigue-related events to rise with each additional patient [27]. In a qualitative study from our group, ICU physicians likewise described decision fatigue as a gradual, context-dependent process [10].

The more striking finding concerns temporal structure: clustered shifts were associated with markedly lower mood disturbance, whilst quiet, uniformly paced shifts showed the least favourable psychometric values. As this group comprised only five shifts, the observation requires replication, and unmeasured differences in case mix, staffing, or baseline mood cannot be excluded as contributing factors. The Job Demands–Resources model and the challenge–hindrance distinction offer one interpretation [28–31]: complex, meaningful clinical decisions act as challenge demands that stimulate engagement, whilst interruptions and administrative burden act as hindrance demands [32–34]. That shift type affected mood but not the decision-fatigue score fits this reading: mood may be more sensitive to the qualitative character of workload, whilst cumulative volume drives fatigue more directly. Consistent with this, a higher decision severity index, reflecting a greater share of complex decisions, was independently associated with lower mood disturbance and lower sleepiness in the adjusted models. Three non-exclusive mechanisms could explain the benefit of clustered work; none of them was measured directly, and they should therefore be regarded as possible explanations rather than demonstrated mediators. Dense decision episodes may induce flow-like states of concentrated, goal-directed activity, which have been linked to better performance and less burnout [35, 36]. The quiet intervals between bursts may act as informal micro-recovery periods not captured by our break measure. And quiet, uniform shifts may provide too little task engagement to prevent mind-wandering, which Killingsworth and Gilbert found to be frequent during undemanding activities and associated with lower momentary happiness [37]. Alternatively, uniformly paced shifts may differ in organisational or interpersonal demands that are not captured by decision counts. Consistent with evidence that mood during a shift reflects lagged and accumulated task exposure rather than overall volume alone [38], mood differences between shift types were already apparent at midday, suggesting that the workload pattern shapes the affective trajectory early rather than only cumulatively.

Breaks, by contrast, showed no clear association with the psychometric outcomes: physicians took on average 2.5 breaks per shift totalling 77.8 min, yet neither the recorded number nor the duration of these periods was associated with any of the three scores. As physicians remained on-site and clinically available throughout, breaks may not have provided sufficient cognitive disengagement to confer measurable recovery benefit, suggesting that recovery quality may matter more than quantity [2].

The exploratory analyses are hypothesis-generating. The descriptive trend towards more conservative scenario responses in busy-clustered shifts is directionally consistent with depletion accounts but statistically inconclusive. Response certainty, by contrast, was lower after shifts with more task interruptions, which may indicate that workflow disruption is related to decision confidence independently of fatigue level. The Decision Severity Score proved practicable for real-time classification. Residents documented nearly twice the proportion of medium or larger decisions compared with consultants (*14.3%* vs. *7.7%*), and the most experienced consultant showed the lowest evening decision-fatigue score and the only negative mean morning-to-evening change. This pattern is consistent with experience buffering against decision fatigue through more automated processing of routine decisions and greater tolerance of clinical uncertainty [10], though it remains strictly anecdotal at *n = 3* consultants.

Although these findings stem from a small, single-centre sample and require replication before informing practice, they point to potentially relevant considerations for clinical work organisation. Shift scheduling might take into account not only total decision volume but also its temporal distribution: in our data, concentrated, meaningful workload appeared better tolerated than uniform, low-intensity load. Organisational interventions protecting uninterrupted clinical activity, such as structured handover protocols and reduction of administrative burden, warrant prospective evaluation and should not yet be considered superior to interventions that reduce overall workload. Future intervention studies could evaluate whether decision-support tools for routine choices reduce the cognitive cost of low-complexity decisions whilst preserving the engagement value of complex clinical work [7, 26], and whether proactive break scheduling with genuine cognitive disengagement is effective as a modifiable intervention [2]. Whether emerging artificial-intelligence-based decision support can eventually absorb part of the routine decision load without introducing new hindrance stressors such as alert burden remains an open question; current intensive-care applications are of low-to-intermediate clinical maturity, with no evidence yet supporting routine deployment [39].

Future research should employ larger samples with objective outcome measures to determine whether the patterns observed here translate into differences in decision quality and patient safety, examine whether modifying temporal workload structure can causally reduce decision fatigue [40, 41], and formally validate the severity score whilst investigating clinical experience as a potential moderator of susceptibility.

### Strengths and limitations

To our knowledge, this is the first study to combine continuous real-time shadowing of ICU physicians across multiple shifts with repeated psychometric assessments and a standardised task, capturing decisions as they occurred rather than by recall. Both burstiness and the Decision Severity Score are novel methodological contributions to decision fatigue research in ICU work, and the study may be viewed as a form of “quantitative ethnography” of intensive care work.

Our study has several limitations. Only five physicians contributed data, and high intraclass correlations indicate that stable between-physician differences dominated the variance, leaving low power at the shift level. The single-centre design and direct recruitment limit generalisability and introduce volunteer bias, and observer presence may have influenced behaviour. The Decision Severity Score was developed *de novo*, was not externally validated, and incorporates physician experience, rendering score-based analyses exploratory; experience levels were not compared inferentially. The median-based shift types formed small, unevenly distributed subgroups, and the mood-score advantage of busy over quiet-smooth shifts did not survive a rank-based sensitivity analysis. Several potential confounders, including patient acuity, staffing, and prior sleep, were not recorded, and the shift-level design precludes causal inference. In keeping with the exploratory design, the findings are best regarded as hypothesis-generating and warrant confirmation in larger studies.

## Conclusion

In this exploratory single-centre study, decision volume and temporal workload structure showed different associations with psychometric outcomes: higher decision rates were associated with greater end-of-shift decision fatigue, whereas greater temporal clustering was associated with lower mood disturbance, a difference already apparent at midday. Mean score changes across the shift were small; more quiet and uniformly paced shifts showed the least favourable psychometric values. A descriptive trend towards more conservative scenario responses in busier shifts is consistent with resource depletion accounts of decision fatigue. Mitigation strategies may need to address the temporal structure of workload in addition to decision volume, preserving meaningful clinical challenge whilst reducing hindrance stressors.

## Supplementary Information

Supplementary material 1

## Data Availability

The datasets generated and/or analysed during the current study are available, in pseudonymised form, in the Supplementary Information files accompanying this article

## Abbreviations

CI: Confidence interval
ICC: Intraclass correlation coefficient
ICU: Intensive care unit
OR: Odds ratio
SD: Standard deviation
SE: Standard error
SOP: Standard operating procedure

## Declarations

### Use of artificial intelligence

During the preparation of this manuscript and its revision, the authors used Claude (Anthropic, versions Sonnet 4.5, 2025, and Fable 5, 2026) for language editing, structural revision, consistency checks, translation of the study scenarios from German into English for the Supplement, and assistance with the preparation and revision of the analysis code. All AI-generated suggestions and code modifications were independently reviewed and verified by the authors. The authors retained full responsibility for the study design, statistical analysis, interpretation, and scientific conclusions. No AI tool was used for the interpretation of results or the generation of scientific conclusions, and no identifiable patient or participant data were entered into the AI systems.
