# Supplementary material for: Decision volume and temporal workload structure as dissociable correlates of fatigue and mood in intensive care physicians: a real-time observational study
Source: Crit Care. 2026 Oct 17;30:534. doi: 10.1186/s13054-026-06249-6 (PMC13679110; doi:10.1186/s13054-026-06249-6)
Supplement: Supplementary file 1 [file 13054_2026_6249_MOESM1_ESM.pdf]

# Supplementary Material

Decision Volume and Temporal Workload Structure as Dissociable Correlates of Fatigue and Mood in Intensive Care Physicians: A Real-Time Observational Study

## Contents


**1 Supplement S1 — Decision Severity Score 2**

**2 Supplement S2 — Standardised Clinical Scenarios 4**

**3 Supplement S3 — Recruitment and Observer Details 35**

**4 Supplement S4 — Sensitivity and Robustness Analyses 37**

**5 Supplement S5 — Shift-Type Descriptive Statistics 40**

**6 Supplement S6 — Scenario Model: Full Results 41**

**7 Supplement S7 — Random Effects, ICC, and Trajectory Model Outputs 42**

**8 Supplement S8 — Model Diagnostics and Unmodelled Temporal Covariates 46**

**9 Supplement S9 — Additional Data Files 50**

**10 Supplement S10 — Per-Shift Data Listing 51**

# 1 Supplement S1 — Decision Severity Score

## Overview

To classify the complexity and potential impact of recorded clinical decisions, a study-specific Decision Severity Score was developed *de novo*, as no validated instrument for rating the severity of individual clinical decisions in the ICU context was available. The score comprises six dimensions — patient risk, decision complexity, consequences, time pressure, evidence base, and physician experience — each rated on a three-point scale, yielding a sum score from 6 to 18. Decisions were classified as small ($S$: 6–8), medium ($M$: 9–14), or large ($L$: 15–18). The score was developed collaboratively by the study team and applied consistently throughout data collection by the trained observer. Formal psychometric validation of the instrument was beyond the scope of this study and represents a direction for future work.

The score was iteratively refined during the one-week pilot phase (February 2025) through discussion within the research team, but scoring was performed by a single observer throughout both the pilot and main data collection; no formal inter-rater reliability assessment (e.g. Cohen's $\kappa$) was conducted. The scoring examples below (Section S1, Scoring Examples) are intended to provide transparency regarding the scoring logic and to facilitate future inter-rater calibration studies.

Table S1: Decision Severity Score. Each of the six dimensions is rated on a three-point scale; the sum score ranges from 6 to 18. Decisions were classified as small ($S$: score 6–8), medium ($M$: score 9–14), or large ($L$: score 15–18).

| Dimension | Criterion | Score |
|---|---|---|
| Risk to the patient | Low | 1 |
| | Moderate but controllable | 2 |
| | High, life-threatening | 3 |
| Complexity of the decision | Simple, standard procedure | 1 |
| | Complex, requires clinical experience | 2 |
| | Highly complex, multiple variables | 3 |
| Consequences of the decision | Short-term, minor | 1 |
| | Minor, reversible, non-critical | 2 |
| | Potentially critical, irreversible | 3 |
| Time pressure | Decision required within 12 h | 1 |
| | Moderate urgency, within 6 h | 2 |
| | Acute need for action, emergency | 3 |
| Evidence and uncertainty | Strong evidence, SOP available | 1 |
| | Inconclusive evidence base | 2 |
| | Unclear or absent evidence base | 3 |
| Experience of the physician | >5 years of ICU experience | 1 |
| | 2–5 years of ICU experience | 2 |
| | 0–2 years of ICU experience | 3 |
| Sum score 6–8 | | Small ($S$) |
| Sum score 9–14 | | Medium ($M$) |
| Sum score 15–18 | | Large ($L$) |

SOP = standard operating procedure.

## Scoring Examples

Three contrasting examples illustrate the scoring logic.

**Small (score = 8):** A first-year resident deciding whether to continue a maintenance glucose infusion in a haemodynamically stable patient with no time pressure and a clear SOP available would score low across most dimensions (patient risk = 1, complexity = 1, consequences = 1, time pressure = 1, evidence = 1, experience = 3), yielding a sum score of 8 and a classification of *small*.

**Medium (score = 10):** The same resident placing a central venous catheter in a young, otherwise healthy patient without time pressure would score higher on complexity (= 2) and consequences (= 2) given the procedural demands and potential for reversible complications such as pneumothorax, resulting in a sum score of 10 and a classification of *medium*.

**Large (score = 15–17):** A senior consultant deciding whether to initiate emergency extracorporeal membrane oxygenation (ECMO) in a patient with refractory septic shock — a decision with high patient risk, multiple interacting variables, acute time pressure, and limited evidence — would score near the maximum on most dimensions (experience = 1), yielding a sum score of 15–17 and a classification of *large*.

# 2 Supplement S2 — Standardised Clinical Scenarios

## Overview

Eight standardised clinical scenarios were used across the observation period, with each participant completing one scenario per shift. All scenarios were developed by three senior ICU consultants (D.L., S.B., M.K.) not otherwise involved in the study and were reviewed for clinical plausibility prior to use. Each decision was classified as category 5 (therapeutic procedure-related) of the clinical decision taxonomy by Ofstad et al. [1] and framed as a binary choice between a Conservative Decision (CD) and an Active Decision (AD). Table S2 summarises the characteristics of each scenario; the full scenario texts are provided in Sections S2.1–S2.8 below.

As consultants and residents hold different levels of clinical responsibility, each scenario comprised two role-specific sets of three binary decision questions: one addressing decisions typically delegated to the supervising consultant (background duties) and one addressing decisions typically made by the resident managing the patient at the bedside (foreground duties). Participants answered the question set corresponding to their role at the time of observation. Table S3 presents the consensus reference answers for both role-specific question sets, developed independently by the three scenario authors.

Where role-specific versions of a scenario differed in clinically relevant parameter values (e.g. fibrinogen in Scenario 1), this was a deliberate feature of scenario design to ensure that the laboratory context was appropriate to the decision posed to each role, rather than an inconsistency.

The scenario topics were selected to reflect the typical case mix of the unit, which regularly manages patients on extracorporeal membrane oxygenation as well as neurosurgical and cardiac surgical patients requiring postoperative intensive care.

Table S2: Characteristics of the eight standardised clinical scenarios. Word counts refer to the full scenario text presented to participants, including decision questions but excluding explanatory notes. Laboratory data counts include routine blood results (Lab), blood gas analyses (BGA), and cerebrospinal fluid results (CSF). All scenarios incorporated radiological findings (chest X-ray, CT, or both) presented as written reports.

| Scenario | Words | Lab | BGA | CSF | Total lab |
|---|---|---|---|---|---|
| 1 | 845 | 32 | 104 | — | 136 |
| 2 | 994 | 36 | 104 | — | 140 |
| 3 | 764 | 68 | 39 | — | 107 |
| 4 | 948 | 58 | 104 | — | 162 |
| 5 | 688 | 33 | 52 | 12 | 97 |
| 6 | 897 | 44 | 39 | — | 83 |
| 7 | 669 | 11 | 65 | — | 76 |
| 8 | 598 | 30 | 26 | — | 56 |
| Mean (SD) | 800 (143) | 39 (18) | 67 (33) | — | 107 (36) |

Lab = routine laboratory values; BGA = blood gas analysis; CSF = cerebrospinal fluid.

### S2.1 — Scenario 1: ECMO Patient Following ECPR for OHCA and Pulmonary Embolism

Two role-specific versions of this scenario were administered: one addressing decisions typically delegated to the supervising consultant (background duties), and one addressing decisions typi-

Table S3: Consensus reference answers for the three binary decisions per scenario, developed independently by the three scenario authors (D.L., S.B., M.K.). Reference answers are given separately for physicians assigned to background duties (consultants) and foreground duties (residents), reflecting differences in role-typical clinical responsibility. CD = Conservative Decision (consistent with the safer/status-quo option); AD = Active Decision. Author = scenario developer.

| Scenario | Topic | Author | Consultants (background) | Residents (foreground) |
|---|---|---|---|---|
| 1 | ECMO | DL | 1. Distal perfusion (CD); 2. Catecholamine weaning (AD); 3. VAV upgrade (AD) | 1. Distal perfusion (CD); 2. Volume administration (AD); 3. Fibrinogen (CD) |
| 2 | ECMO | DL | 1. Therapeutic hypothermia (AD); 2. Iloprost (AD); 3. Levetiracetam (CD) | 1. Anaconda cannula adjustment (AD); 2. Volume administration (AD); 3. Levetiracetam (CD) |
| 3 | CVC exchange | MK | 1. De-escalation of antibiotics (AD); 2. Prone positioning (AD); 3. Shaldon catheter + dialysis (AD) | 1. Furosemide perfusion (AD); 2. Bronchoscopy (AD); 3. Shaldon catheter + dialysis (AD) |
| 4 | Cranial CT | MK | 1. Catheter exchange (AD); 2. Extubation (AD); 3. Discontinuation of antibiotics (AD) | 1. Nutrition (AD); 2. Extubation (AD); 3. Arterial line (CD) |
| 5 | Positioning/Ventilation | MK | 1. Catheter exchange (AD); 2. Resumption of antibiotics (CD); 3. Chest X-ray (CD) | 1. Catheter exchange (AD); 2. Red cell transfusion (CD); 3. Arterial line (CD) |
| 6 | Extubation | SB | 1. Furosemide perfusion (AD); 2. CT thorax (AD); 3. Red cell transfusion (AD) | 1. Furosemide bolus (AD); 2. CT thorax (AD); 3. Red cell transfusion (AD) |
| 7 | Dialysis | SB | 1. Escalation of antibiotics (CD); 2. Discontinuation of prone positioning (AD); 3. CT pulmonary angiogram (AD) | 1. Albumin administration (AD); 2. Discontinuation of prone positioning (AD); 3. CT pulmonary angiogram (AD) |
| 8 | Transfusion | SB | 1. Red cell transfusion (CD); 2. Diuretic (AD); 3. Transfer (AD) | 1. Red cell transfusion (CD); 2. Potassium perfusion (CD); 3. Furosemide perfusion (AD) |

cally made by the resident managing the patient at the bedside (foreground duties). The patient profile and Day 1 status were identical across both versions; the three decision questions and selected laboratory values differed by role (see Table S3).

**Patient Profile (both versions)**

- Female, 42 years, 68 kg
- **Diagnosis:** out-of-hospital cardiac arrest (OHCA; cumulative 48 min CPR) due to pulseless electrical activity, secondary to fulminant central pulmonary embolism; pre-hospital thrombolysis and subsequent extracorporeal cardiopulmonary resuscitation (ECPR) on scene
- **Past medical history:** nicotine use, recent immobilisation following varicose vein surgery with concurrent flu-like illness (possible influenza), oral contraceptive use
- **Current status, Day 1:**
  - VA-ECMO (right femoral): flow 2.9 L/min, sweep gas $FiO_2$ 0.6 at 3 L/min
  - Status post initial diagnostic work-up and successful FlowTriever® thrombectomy
  - **Medication:** noradrenaline 2.1 mg/h, dobutamine 25 mg/h, vasopressin 2 IU/h, heparin 800 IU/h, propofol 250 mg/h, sufentanil 30 $\mu$g/h, piperacillin/tazobactam 4.5 g three times daily
  - BP 75/70 mmHg, $SpO_2$ 92 %, HR 124/min, sinus rhythm, multiple polymorphic ventricular ectopics
  - Ventilation: $FiO_2$ 70 %, PEEP 14 mbar, peak pressure 30 mbar
  - Core temperature 35.2 °C
  - Near-infrared spectroscopy (NIRS), lower leg: 32 (cannulated leg, arterial and venous cannulae) vs. 47 (non-cannulated leg)
- **Additional findings:** mild nasal bleeding following an unsuccessful attempt at nasogastric tube placement

**Laboratory Parameters, Day 1**

**Arterial Blood Gas, Day 1**

**Whole-Body CT, Day 1 (Initial Imaging)**

A massive central pulmonary embolism is demonstrated, with filling defects in both main pulmonary arterial branches and a substantial thrombus burden, particularly in the right pulmonary artery. The right heart is markedly dilated (RV/LV ratio 1.9), with leftward bowing of the interventricular septum consistent with acute pressure overload. The pulmonary veins are normally opacified. A discrete area of consolidation is noted in the right dorsobasal lung, consistent with recent aspiration; no features typical of pneumonia are seen. Small bilateral pleural effusions are present. **Foreign material:** endotracheal tube in satisfactory position; right femoral VA-ECMO cannulae, with both arterial and venous limbs appropriately placed. Contrast opacification of the

| Parameter | Value |
|---|---|
| Hb (g/dL) | 9.4 |
| Hct (%) | 28 |
| Platelets (/nL) | 74 |
| Leukocytes (/nL) | 18.2 |
| CRP (mg/L) | 48 |
| Procalcitonin (ng/mL) | 0.9 |
| INR | 2.1 |
| aPTT (s) | 86 |
| Fibrinogen (mg/dL) | 110 (Consultant version) / 210 (Resident version) |
| D-dimer (mg/L) | >20 |
| Creatinine (mg/dL) | 2.1 |
| Urea (mg/dL) | 75 |
| Bilirubin (mg/dL) | 2.8 |
| AST (U/L) | 410 |
| ALT (U/L) | 365 |
| LDH (U/L) | 1120 |

**Day 1 laboratory parameters. Fibrinogen differed between the two role-specific scenario versions (see text).**

| Parameter | 08:00 | 12:00 | 16:00 | 20:00 |
|---|---|---|---|---|
| pH | 7.19 | 7.26 | 7.31 | 7.32 |
| $PaO_2$ (mmHg) | 59 | 61 | 60 | 65 |
| $PaCO_2$ (mmHg) | 52 | 48 | 44 | 43 |
| $HCO_3^-$ (mmol/L) | 19 | 20 | 21 | 22 |
| Base excess (mmol/L) | −6 | −5 | −4 | −3 |
| $SaO_2$ (%) | 89 | 92 | 91 | 90 |
| Lactate (mg/dL) | 77 | 70 | 65 | 62 |
| $Na^+$ (mmol/L) | 144 | 144 | 145 | 145 |
| $K^+$ (mmol/L) | 4.6 | 4.5 | 4.4 | 4.3 |
| $iCa^{2+}$ (mmol/L) | 1.08 | 1.07 | 1.07 | 1.06 |
| $Cl^-$ (mmol/L) | 108 | 109 | 109 | 110 |
| Glucose (mg/dL) | 210 | 205 | 200 | 195 |

**Day 1 arterial blood gas series. Central venous oxygen saturation ($ScvO_2$, %): 08:00 = 52; 12:00 = 55; 16:00 = 58; 20:00 = 60.**

right (cannulated) leg is present but markedly reduced compared with the left, consistent with haemodynamic compromise from the cannulation. No evidence of relevant resuscitation-related trauma. No intracerebral haemorrhage. Abdominal organs show no acute pathology.

**Chest CT, Day 1 (Follow-up After Intervention)**

Following FlowTriever® thrombectomy, no central thrombi remain demonstrable; the central pulmonary arteries are recanalised. The right heart remains enlarged, though septal bowing is somewhat reduced. Parenchyma: unchanged aspiration-associated consolidation in the right dorsobasal lung, with no clear evidence of pneumonia. Small bilateral pleural effusions persist. **Foreign material:** endotracheal tube and ECMO cannulae unchanged and appropriately positioned. Additionally, two central venous catheters via the right internal jugular vein, with satisfactory intrathoracic position.

**Decision Questions — Consultant Version (Background Duties)**

**Question 1.** Should the patient immediately receive an antegrade distal perfusion cannula in the cannulated leg? Please select one option and briefly justify your decision.

- Yes, the patient requires a distal perfusion cannula.
- No, the patient does not require a distal perfusion cannula.

On a scale of 1–5, how confident are you in this decision (1 = very uncertain, 5 = very confident)?

**Question 2.** Should dobutamine be switched to adrenaline without delay? Please select one option and briefly justify your decision.

- Yes, dobutamine is insufficient; I switch to adrenaline, or add adrenaline to the current regimen.
- No, I administer a fluid bolus first and reassess.

On a scale of 1–5, how confident are you in this decision?

*Day 2 update (Consultant version):* VA-ECMO unchanged (2.9 L/min, sweep $FiO_2$ 0.6 at 3 L/min). Medication: noradrenaline 2.1 mg/h, dobutamine 25 mg/h, vasopressin 2 IU/h, heparin 1100 IU/h, propofol 250 mg/h, sufentanil 40 $\mu$g/h, piperacillin/tazobactam 4.5 g three times daily. BP 85/50 mmHg, $SpO_2$ 88 %, HR 110/min, sinus rhythm. Ventilation: $FiO_2$ 95 %, PEEP 17 mbar, peak pressure 30 mbar. Core temperature 36.2 °C. NIRS lower leg: 45 (cannulated) vs. 47 (non-cannulated).

Day 2 laboratory and blood gas trends showed a rise in CRP (118 mg/L) and procalcitonin (3.8 ng/mL), with progressive bilateral basal-predominant consolidations on chest CT, consistent with aspiration pneumonia evolving into more extensive consolidation, and multiple adjacent atelectatic areas with moderate bilateral pleural effusions. Foreign material (endotracheal tube, right femoral ECMO cannulae, right internal jugular central venous catheter) remained appropriately positioned.

**Question 3.** Given the pulmonary findings, an additional jugular cannula should be inserted and the current VA-ECMO escalated to VAV-ECMO. Please select one option and briefly justify your decision.

- Yes, an upgrade to VAV configuration makes good sense.
- No, I prioritise drainage of the pleural effusions and kinetic therapy (full prone positioning).

On a scale of 1–5, how confident are you in this decision?

**Decision Questions — Resident Version (Foreground Duties)**

**Question 1.** (Identical to the consultant version above: antegrade distal perfusion cannula.)

*Day 2 update (Resident version):* VA-ECMO unchanged (2.9 L/min, sweep $FiO_2$ 0.6 at 3 L/min). Medication: noradrenaline 2.1 mg/h, dobutamine 25 mg/h, vasopressin 2 IU/h, heparin 1100 IU/h, propofol 250 mg/h, sufentanil 40 $\mu$g/h, piperacillin/tazobactam 4.5 g three times daily. BP 85/50 mmHg, $SpO_2$ 88 %, HR 110/min, sinus rhythm. Ventilation: $FiO_2$ 95 %, PEEP 17 mbar, peak pressure 30 mbar. Core temperature 36.2 °C. NIRS lower leg: 45 (cannulated) vs. 47 (non-cannulated). Fibrinogen on Day 2: 140 mg/dL.

Day 2 chest CT findings were identical to those described above (progressive aspiration-associated consolidation, moderate bilateral effusions, foreign material unchanged).

**Question 2.** *The ECMO repeatedly triggers a negative-pressure ("suck-down") alarm.* Should a fluid bolus be administered without delay? Please select one option and briefly justify your decision.

- Yes, a fluid bolus makes good sense.
- No, I reposition the patient to prevent further suck-down events.

On a scale of 1–5, how confident are you in this decision?

**Question 3.** Given the bleeding tendency from the nose following nasogastric tube placement, the patient requires fibrinogen.

- Yes, I administer, e.g., 2 g of fibrinogen.
- No, I opt for a watch-and-wait approach.

On a scale of 1–5, how confident are you in this decision?

## S2.2 — Scenario 2: ECMO Patient Following ECPR for OHCA and STEMI

As in Scenario 1, two role-specific versions were administered, addressing decisions typically delegated to the supervising consultant (background duties) and decisions typically made by the resident managing the patient at the bedside (foreground duties). The patient profile and Day 1 status were identical across both versions; the three decision questions and selected laboratory values differed by role (see Table S3).

**Patient Profile (both versions)**

- Male, 72 years, 98 kg
- **Diagnosis:** out-of-hospital cardiac arrest (OHCA; cumulative 53 min CPR) due to initial ventricular fibrillation, secondary to ST-elevation myocardial infarction (proximal occlusion of the ramus circumflexus), and subsequent extracorporeal cardiopulmonary resuscitation (ECPR) on scene
- **Past medical history:** arterial hypertension, non-insulin-dependent diabetes mellitus, hypercholesterolaemia, COPD, nicotine use
- **Current status, Day 1:**
  - VA-ECMO (right femoral)
  - Status post percutaneous coronary intervention with implantation of two drug-eluting stents using the reversed-crush technique in the left main stem and proximal circumflex artery
  - NIRS, lower leg: 52 (cannulated leg, arterial and venous cannulae) vs. 56 (non-cannulated leg)
  - **Physical examination:** on admission, sedated (RASS $-4$); pupils mid-sized, isocoric, sluggish light reflex bilaterally; intermittent myoclonus of the arms and legs. Heart sounds normal and rhythmic, tachycardic. Circulation moderately stable on catecholamine support (noradrenaline 1.1 mg/h, dobutamine 30 mg/h, vasopressin 2 IU/h) and VA-ECMO (flow 3.7 L/min, sweep gas $FiO_2$ 0.6 at 2 L/min). Lungs: endotracheally intubated, tube in correct position, controlled ventilation ($FiO_2$ 60 %, PEEP 10 mbar, peak pressure 17 mbar); bilateral coarse crackles with expiratory wheeze. Abdomen soft, non-tender, bowel sounds sparse. Periphery cool, mild bilateral lower-leg oedema, core temperature 36.3 °C.
  - **Further medication:** tirofiban 0.9 mg/h (for 24 h), heparin 400 IU/h, propofol 400 mg/h, sufentanil 40 $\mu$g/h, piperacillin/tazobactam 4.5 g three times daily (dosed for presumed aspiration)
  - BP 85/60 mmHg, $SpO_2$ 94 %, HR 104/min, sinus rhythm, multiple polymorphic ventricular ectopics
- **Additional findings:** mild nasal bleeding following nasogastric tube placement. The patient repeatedly requires additional sedation boluses during repositioning and tracheal suctioning, and intermittently bucks against the endotracheal tube, causing desaturation episodes.

| Parameter | Value |
|---|---|
| Hb (g/dL) | 12.4 |
| Hct (%) | 34 |
| Platelets (/nL) | 344 |
| Leukocytes (/nL) | 13.2 |
| CRP (mg/L) | 32 |
| Procalcitonin (ng/mL) | 0.9 |
| INR | 1.6 |
| aPTT (s) | 86 |
| Fibrinogen (mg/dL) | 410 (Resident version) / 210 (Consultant version) |
| D-dimer (mg/L) | 2.0 |
| Creatinine (mg/dL) | 2.1 |
| Urea (mg/dL) | 75 |
| Bilirubin (mg/dL) | 1.8 |
| AST (U/L) | 410 |
| ALT (U/L) | 365 |
| LDH (U/L) | 1120 |
| NSE (ng/mL) | 52 |
| CK (U/L) | 4950 |
| CK-MB (U/L) | 280 |

**Day 1 laboratory parameters. Fibrinogen differed between the two role-specific scenario versions (see text). LDH was reported twice in the original instrument (1120 and 1650 U/L); the lower value, corresponding to the routine chemistry panel, is reported here.**

| Parameter | 08:00 | 12:00 | 16:00 | 20:00 |
|---|---|---|---|---|
| pH | 7.12 | 7.22 | 7.28 | 7.32 |
| $PaO_2$ (mmHg) | 58 | 62 | 66 | 72 |
| $PaCO_2$ (mmHg) | 55 | 50 | 46 | 48 |
| $HCO_3^-$ (mmol/L) | 18 | 20 | 21 | 22 |
| Base excess (mmol/L) | −8 | −6 | −5 | −3 |
| $SaO_2$ (%) | 86 | 89 | 91 | 93 |
| Lactate (mg/dL) | 88 | 76 | 70 | 65 |
| $Na^+$ (mmol/L) | 142 | 142 | 143 | 143 |
| $K^+$ (mmol/L) | 4.8 | 4.7 | 4.6 | 4.9 |
| $iCa^{2+}$ (mmol/L) | 1.05 | 1.05 | 1.06 | 1.06 |
| $Cl^-$ (mmol/L) | 110 | 111 | 111 | 112 |
| Glucose (mg/dL) | 210 | 205 | 200 | 195 |

**Day 1 arterial blood gas series. Central venous oxygen saturation ($ScvO_2$, %): 08:00 = 52; 12:00 = 55; 16:00 = 58; 20:00 = 60.**

**Laboratory Parameters, Day 1**

**Arterial Blood Gas, Day 1**

**Whole-Body CT, Day 1 (Initial Imaging)**

No central or segmental pulmonary embolism is demonstrated. The right heart is mildly dilated, with discreet leftward septal bowing. The lung parenchyma shows bilateral basal-predominant consolidated areas, more pronounced on the right, consistent with aspiration-related infiltrates. Additional features of chronic obstructive pulmonary disease are noted, with generalised hyperinflation, irregular bronchial wall thickening, and multiple bullous emphysematous changes in both upper lobes. Bilateral multiple rib fractures are present, predominantly anterior and posterior on the right, without evidence of haemothorax or pneumothorax. Small bilateral pleural effusions. Abdominal organs within the field of view show no acute pathology. No evidence of acute intracerebral pathology. **Foreign material:** endotracheal tube in correct position; right femoral VA-ECMO cannulae (arterial and venous) and a right internal jugular central venous catheter, all appropriately placed.

**Echocardiography, Day 1**

Left ventricular function is markedly impaired with a globally hypokinetic pattern, estimated ejection fraction approximately 20 %. The left ventricle is overall dilated, with hypertrophy of the basal septal and posterior walls. Moderate mitral regurgitation and moderate tricuspid regurgitation are present. The aortic valve is sclerotic with mild stenosis, without haemodynamically relevant regurgitation. Right ventricular function is preserved. The right atrium is dilated. Estimated systolic pulmonary artery pressure is approximately 50 mmHg. No pericardial effusion. No evidence of intracardiac thrombus.

**Decision Questions — Consultant Version (Background Duties)**

**Question 1.** Should the patient be cooled to a target temperature of 34.0 °C for 24 hours? Please select one option and briefly justify your decision.

- Yes, I cool the patient for 24 hours.
- No, the patient should be kept normothermic.

On a scale of 1–5, how confident are you in this decision (1 = very uncertain, 5 = very confident)?

**Question 2.** Should the patient receive inhaled iloprost (e.g. 5 $\mu$g six times daily)? Please select one option and briefly justify your decision.

- Yes, this makes good sense.
- No, I wait and reassess.

On a scale of 1–5, how confident are you in this decision?

*Day 2 update (Consultant version):* VA-ECMO flow 3.9 L/min, sweep gas $FiO_2$ 0.6 at 3 L/min. Medication: noradrenaline 1.2 mg/h, dobutamine 30 mg/h, vasopressin 2 IU/h, heparin 1100 IU/h,

propofol 400 mg/h, sufentanil 40 μg/h, piperacillin/tazobactam 4.5 g three times daily. BP 85/50 mmHg, $SpO_2$ 88 %, HR 110/min, sinus rhythm. Ventilation: $FiO_2$ 95 %, PEEP 17 mbar, peak pressure 30 mbar. NIRS lower leg: 45 (cannulated) vs. 47 (non-cannulated). Platelets fell to 62/nL; fibrinogen rose to 540 mg/dL.

**Question 3.** Given the patient's ongoing myoclonus, levetiracetam therapy is indicated.

- Yes, I prescribe levetiracetam 1 g i.v. twice daily.
- No, I opt for a watch-and-wait approach.

On a scale of 1–5, how confident are you in this decision?

**Decision Questions — Resident Version (Foreground Duties)**

**Question 1.** Should the patient be sedated with inhaled isoflurane (via AnaConDa device)? Please select one option and briefly justify your decision.

- Yes, switch the patient from propofol to isoflurane sedation.
- No, I deepen sedation by increasing the propofol dose.

On a scale of 1–5, how confident are you in this decision?

*Day 2 update (Resident version):* VA-ECMO flow 3.9 L/min, sweep gas $FiO_2$ 0.6 at 3 L/min. Medication: noradrenaline 1.2 mg/h, dobutamine 30 mg/h, vasopressin 2 IU/h, heparin 1100 IU/h, propofol 500 mg/h or isoflurane, sufentanil 40 μg/h, furosemide 20 mg/h, piperacillin/tazobactam 4.5 g three times daily. BP 85/50 mmHg, $SpO_2$ 88 %, HR 110/min, sinus rhythm. Ventilation: $FiO_2$ 95 %, PEEP 17 mbar, peak pressure 30 mbar. NIRS lower leg: 45 (cannulated) vs. 47 (non-cannulated). The ECMO repeatedly triggers a negative-pressure ("suck-down") alarm. Platelets fell to 62/nL; fibrinogen rose to 540 mg/dL.

**Question 2.** Should the patient receive a fluid bolus? Please select one option and briefly justify your decision.

- Yes, I administer e.g. 500–1000 mL of balanced crystalloid.
- No, I attempt repositioning and reassess.

On a scale of 1–5, how confident are you in this decision?

**Question 3.** Given the patient's ongoing myoclonus, levetiracetam therapy is indicated.

- Yes, I administer levetiracetam 1 g i.v. twice daily.
- No, I opt for a watch-and-wait approach.

On a scale of 1–5, how confident are you in this decision?

## S2.3 — Scenario 3: ARDS Following Community-Acquired Pneumonia (Part I)

Scenarios 3 and 4 describe the same patient followed across two consecutive observation days; Scenario 3 covers Days 1–5 of the clinical course, and Scenario 4 (Section S2.4) continues from Day 6 to Day 14. As in the preceding scenarios, two role-specific sets of three decision questions were administered (consultant/background and resident/foreground; see Table S3).

### Patient Profile

- Male, 68 years, 178 cm, 101 kg (BMI 32 $kg/m^2$, obesity class I)
- **Reason for admission:** community-acquired lobar pneumonia
- **Past medical history:**
  - Arterial hypertension (treated, mostly ramipril/amlodipine)
  - Type 2 diabetes mellitus (oral antidiabetics, most recent HbA1c 7.8 %)
  - COPD GOLD III, ex-smoker with 45 pack-years, on inhaled therapy (LABA/LAMA/ICS)
  - Obesity class I

### Chest X-ray on Admission

Posteroanterior chest radiograph. Hyperinflated lungs with flattened diaphragms and increased radiolucency. The right basal-dorsal region shows a homogeneous, segmental-to-lobar opacification with positive air bronchogram, consistent with lobar pneumonia. No infiltrate on the left. Heart size normal, central vascular markings prominent, no evidence of pleural effusion or pneumothorax.

### Clinical Course, Days 1–3

**Days 1–2:** Admission with severe pneumonia and hypoxaemia ($PaO_2/FiO_2 \approx 180$); initially high-flow nasal cannula, then non-invasive ventilation, with rapidly progressive respiratory failure. Blood cultures and tracheal aspirate sent. Piperacillin/tazobactam and azithromycin started.

**Day 3:** Intubation using videolaryngoscopy and bougie due to a short neck (Cormack–Lehane grade III); invasive ventilation (PEEP 12 $cmH_2O$, $FiO_2$ 0.8). Haemodynamic instability with septic shock, requiring noradrenaline up to 1.8 mg/h and vasopressin 1 IU/h. In the afternoon, microbiology results return: *Streptococcus pneumoniae* in tracheal aspirate and blood cultures, sensitive to ceftriaxone.

### Laboratory Parameters, Days 1–3

### Decision Question 1 — Consultant Version (Background Duties)

**Question 1.** Should antibiotic therapy be de-escalated to ceftriaxone? Please select one option and briefly justify your decision.

- Yes, I de-escalate to ceftriaxone.

| Parameter | Day 1 | Day 2 | Day 3 |
|---|---|---|---|
| Hb (g/dL) | 9.8 | 9.2 | 8.5 |
| Hct (%) | 30 | 28 | 26 |
| Platelets (/nL) | 290 | 250 | 210 |
| Leukocytes (/nL) | 13.5 | 15.2 | 18.0 |
| CRP (mg/L) | 160 | 220 | 280 |
| Procalcitonin (ng/mL) | 3.5 | 8.2 | 15.0 |
| INR | 1.3 | 1.4 | 1.3 |
| aPTT (s) | 29 | 32 | 31 |
| Creatinine (mg/dL) | 1.9 | 2.8 | 3.6 |
| Urea (mg/dL) | 70 | 90 | 115 |
| Bilirubin (mg/dL) | 1.4 | 1.8 | 2.4 |
| AST (U/L) | 160 | 280 | 480 |
| ALT (U/L) | 130 | 210 | 370 |
| LDH (U/L) | 750 | 1100 | 1650 |

- No, I wait and reassess the situation the following day.

On a scale of 1–5, how confident are you in this decision (1 = very uncertain, 5 = very confident)?

### Decision Question 1 — Resident Version (Foreground Duties)

*Additional information (Resident version):* The patient's urine output decreases over the course of the day, with hourly volumes around 40 mL/h; the current fluid balance is +250 mL.

**Question 1.** Should the patient receive continuous furosemide via syringe driver? Please select one option and briefly justify your decision.

- Yes, I start a furosemide infusion.
- No, I wait and reassess the situation the following day.

On a scale of 1–5, how confident are you in this decision?

### Clinical Course, Day 5

Development of moderate-to-severe ARDS ($PaO_2/FiO_2 < 100$). Bronchoalveolar lavage performed. Circulation remains compromised: noradrenaline 1.7 mg/h, vasopressin 1 IU/h. Diuresis declining, 20 mL/h on a furosemide infusion at 20 mg/h.

**Chest CT:** Extensive bilateral confluent ground-glass opacities and partly consolidated areas with a ventro-dorsal gradient, more pronounced basally. Near-complete consolidation dorsally, with relative ventral lucency preserved. Small bilateral pleural effusions. No evidence of central pulmonary embolism. Mediastinal structures unremarkable, no pathologically enlarged lymph nodes. **Foreign material:** endotracheal tube in correct position, tip a few centimetres above the carina; right internal jugular central venous catheter with tip at the superior vena cava; nasogastric tube in gastric position.

Ventilation (Day 5): BiVent mode, $FiO_2$ 80 %, PEEP 14 mbar, plateau pressure 30 mbar.

| Parameter | Day 4 | Day 5 |
|---|---|---|
| Hb (g/dL) | 8.4 | 8.2 |
| Hct (%) | 25 | 25 |
| Platelets (/nL) | 200 | 190 |
| Leukocytes (/nL) | 16.8 | 17.2 |
| CRP (mg/L) | 250 | 260 |
| Procalcitonin (ng/mL) | 11.8 | 12.0 |
| INR | 1.3 | 1.4 |
| aPTT (s) | 31 | 32 |
| Creatinine (mg/dL) | 3.7 | 4.2 |
| Urea (mg/dL) | 144 | 195 |
| Bilirubin (mg/dL) | 2.0 | 2.2 |
| AST (U/L) | 400 | 420 |
| ALT (U/L) | 290 | 310 |

| Parameter | 12:00 | 14:00 | 16:00 |
|---|---|---|---|
| pH | 7.29 | 7.24 | 7.21 |
| $PaO_2$ (mmHg) | 78 | 65 | 63 |
| $PaCO_2$ (mmHg) | 52 | 56 | 58/59[a] |
| $HCO_3^-$ (mmol/L) | 23 | 22 | 22 |
| Base excess (mmol/L) | −3 | −4 | −5 |
| $SaO_2$ (%) | 91 | 89 | 87 |
| Lactate (mg/dL) | 12 | 14 | 15 |
| $Na^+$ (mmol/L) | 141 | 142 | 142 |
| $K^+$ (mmol/L) | 4.8 | 5.2 | 5.8 |
| $iCa^{2+}$ (mmol/L) | 1.06 | 1.07 | 1.07 |
| $Cl^-$ (mmol/L) | 110 | 111 | 111 |
| Glucose (mg/dL) | 180 | 185 | 190 |

**Day 5 arterial blood gas series. [a]$PaCO_2$ at 16:00 was recorded as 58 mmHg in the consultant version and 59 mmHg in the resident version of the instrument.**

**Decision Questions 2–3 — Consultant Version (Background Duties)**

**Question 2.** Should the patient be turned to the prone position today? Please select one option and briefly justify your decision.

- Yes, I order prone positioning for 16 hours.
- No, I wait and reassess first thing tomorrow morning.

On a scale of 1–5, how confident are you in this decision?

**Question 3.** Should the patient receive a Shaldon catheter and start dialysis today?

- Yes, the patient should receive a Shaldon catheter and dialysis today.
- No, I wait and reassess first thing tomorrow morning.

On a scale of 1–5, how confident are you in this decision?

**Decision Questions 2–3 — Resident Version (Foreground Duties)**

**Question 2.** Should the patient undergo bronchoscopy today to aspirate potential secretions? Please select one option and briefly justify your decision.

- Yes, I bronchoscope the patient today.
- No, I wait and reassess first thing tomorrow morning.

On a scale of 1–5, how confident are you in this decision?

**Question 3.** Should the patient receive a Shaldon catheter and start dialysis today? *(Identical question and options to the consultant version above.)* On a scale of 1–5, how confident are you in this decision?

## S2.4 — Scenario 4: ARDS Following Community-Acquired Pneumonia (Part II)

Scenario 4 continues directly from Scenario 3 (Section S2.3), describing the same patient's course from Day 6 to Day 14.

### Clinical Course, Day 6

Anuria; continuous renal replacement therapy (CVVHDF) initiated. Slight improvement in oxygenation with prone positioning. In the afternoon, microbiology results from the bronchoalveolar lavage and blood cultures taken on Day 4 return: numerous Gram-negative rods (*Pseudomonas aeruginosa*), few leukocytes; susceptibility testing pending. Antibiotic therapy is empirically escalated to piperacillin/tazobactam and ciprofloxacin. The patient remains catecholamine-dependent (noradrenaline 1.5 mg/h; vasopressin 1 IU/h in the resident version).

*Additional information (Resident version):* The patient had 300 mL of gastric reflux.

| Parameter | Day 6 |
|---|---|
| Hb (g/dL) | 8.0 |
| Hct (%) | 24 |
| Platelets (/nL) | 170 |
| Leukocytes (/nL) | 19.0 |
| CRP (mg/L) | 320 |
| Procalcitonin (ng/mL) | 15.0 |
| INR | 1.5 |
| aPTT (s) | 34 |
| Creatinine (mg/dL) | 3.5 |
| Urea (mg/dL) | 110 |
| Bilirubin (mg/dL) | 2.5 |
| AST (U/L) | 440 |
| ALT (U/L) | 330 |

| Parameter | 08:00 | 11:00 | 14:00 | 16:00 | 18:00 |
|---|---|---|---|---|---|
| pH | 7.23 | 7.26 | 7.29 | 7.32 | 7.35 |
| $PaO_2$ (mmHg) | 60 | 64 | 70 | 71 | 72 |
| $PaCO_2$ (mmHg) | 58 | 55 | 52 | 50 | 52 |
| $HCO_3^-$ (mmol/L) | 22 | 23 | 23 | 24 | 24 |
| Base excess (mmol/L) | −4 | −3 | −2 | −1 | −1 |
| $SaO_2$ (%) | 88 | 90 | 92 | 94 | 95 |
| Lactate (mg/dL) | 14 | 12 | 11 | 10 | 9 |
| $Na^+$ (mmol/L) | 142 | 142 | 143 | 143 | 143 |
| $K^+$ (mmol/L) | 5.4 | 5.0 | 4.7 | 4.5 | 4.3 |
| $iCa^{2+}$ (mmol/L) | 1.07 | 1.07 | 1.08 | 1.08 | 1.09 |
| $Cl^-$ (mmol/L) | 111 | 111 | 110 | 110 | 109 |
| Glucose (mg/dL) | 180 | 175 | 170 | 165 | 160 |

**Day 6 arterial blood gas series.**

### Decision Question 1 — Consultant Version (Background Duties)

**Question 1.** In addition to escalating antibiotic therapy, should the central catheters be exchanged? Please select one option and briefly justify your decision.

- Yes, the catheters need to be exchanged.
- No, I wait and reassess first thing tomorrow morning.

On a scale of 1–5, how confident are you in this decision (1 = very uncertain, 5 = very confident)?

**Decision Question 1 — Resident Version (Foreground Duties)**

**Question 1.** Should enteral feeding be continued? Please select one option and briefly justify your decision.

- Yes, enteral feeding can be continued.
- No, I pause it and reassess the situation first thing tomorrow morning.

On a scale of 1–5, how confident are you in this decision?

**Clinical Course, Days 6–14**

**Days 6–10:** Prone positioning continued in several 16-hour cycles, with marked improvement in oxygenation ($PaO_2/FiO_2$ rises from 120 to approximately 180–200). Ventilation remains controlled, with $FiO_2$ gradually reducible to 0.50–0.55. Haemodynamically, the patient remains noradrenaline-dependent (≈1.5 mg/h on Day 6), with stepwise stabilisation. Days 8–10: circulation increasingly stable, noradrenaline decreasing to 0.8 mg/h, vasopressin discontinued.

**Day 9:** GENIUS dialysis (daily intermittent haemodialysis) initiated. First signs of spontaneous diuresis (≈200–400 mL/24 h). Electrolytes stabilising; urea and creatinine beginning to decline slightly.

**Day 10 — follow-up chest X-ray:** Supine chest radiograph. Endotracheal tube unchanged and correctly positioned. Central venous/Shaldon catheter via the right internal jugular vein in correct position. Bilateral regressing infiltrates, predominantly basal, with residual ground-glass changes in the right lower zone. Costophrenic angles largely free, no major effusions. Heart size normal. No pneumothorax.

**Day 11:** Patient supine again for 24 h, oxygenation stable. Ventilation: $FiO_2$ 0.45, PEEP 10 $cmH_2O$. Catecholamines further reduced, noradrenaline <0.5 mg/h, haemodynamically stable, lactate normal.

**Day 12 — follow-up chest X-ray:** Supine chest radiograph. Endotracheal tube unchanged and correctly positioned. Central venous catheter satisfactory. Both lungs largely cleared, with only discrete residual perihilar opacities. No remaining consolidations, no relevant pleural effusions. Diaphragms well delineated.

**Day 12:** Dialysis weaning trial started following GENIUS therapy. Spontaneous diuresis increasing (800 mL/24 h). Renal parameters rising slightly but without critical derangement: creatinine ≈2.8 mg/dL, urea ≈95 mg/dL. Fluid balance even, electrolytes stable.

**Day 14 (current):** The patient has been undergoing a spontaneous awakening trial since the morning. With sedation reduced, he shows adequate spontaneous breathing in CPAP mode ($FiO_2$ 40 %, PEEP 8 mbar). Copious tracheobronchial secretions requiring regular suctioning.

The patient coughs adequately on endotracheal stimulation and can partially mobilise secretions himself. Neurologically, he opens his eyes to voice, is weak but cooperative, and follows simple commands with delay. *(Resident version additionally notes: he is now nearly catecholamine-free, noradrenaline 0.3 mg/h.)* In the context of the prolonged ICU course, clinical signs of critical illness polyneuropathy/myopathy (CIP/CIM) with generalised muscle weakness are evident.

| Parameter | Day 12 | Day 13 | Day 14 |
|---|---|---|---|
| Hb (g/dL) | 9.0 | 8.5 | 8.0 |
| Hct (%) | 27 | 26 | 24 |
| Platelets (/nL) | 210 | 240 | 260 |
| Leukocytes (/nL) | 15.2 | 12.8 | 10.5 |
| CRP (mg/L) | 180 | 140 | 80 |
| Procalcitonin (ng/mL) | 5.6 | 2.8 | 1.2 |
| INR | 1.4 | 1.5 | 1.3 |
| aPTT (s) | 38 | 38 | 34 |
| Fibrinogen (mg/dL) | 330 | 380 | 430 |
| D-dimer (mg/L) | 4.6 | 3.0 | 1.9 |
| Creatinine (mg/dL) | 3.3 | 2.5 | 2.0 |
| Urea (mg/dL) | 115 | 90 | 70 |
| Bilirubin (mg/dL) | 2.5 | 2.0 | 1.5 |
| AST (U/L) | 480 | 300 | 180 |
| ALT (U/L) | 370 | 250 | 160 |

| Parameter | 08:00 | 12:00 | 16:00 |
|---|---|---|---|
| pH | 7.31 | 7.34 | 7.36 |
| $PaO_2$ (mmHg) | 78 | 76 | 74 |
| $PaCO_2$ (mmHg) | 48 | 45 | 44 |
| $HCO_3^-$ (mmol/L) | 23 | 24 | 24 |
| Base excess (mmol/L) | −2 | −1 | 0 |
| $SaO_2$ (%) | 94 | 93 | 93 |
| Lactate (mg/dL) | 8 | 12 | 10 |
| $Na^+$ (mmol/L) | 141 | 141 | 142 |
| $K^+$ (mmol/L) | 4.6 | 4.5 | 4.4 |
| $iCa^{2+}$ (mmol/L) | 1.07 | 1.08 | 1.09 |
| $Cl^-$ (mmol/L) | 109 | 109 | 110 |
| Glucose (mg/dL) | 185 | 178 | 170 |

**Day 14 arterial blood gas series.**

**Chest X-ray (Day 14):** Supine chest radiograph. Foreign material unchanged and correctly positioned. Bilateral discrete basal opacities consistent with small pleural effusions and residual infiltrates. No extensive consolidations, no new infiltrates. No evidence of pneumothorax.

### Decision Questions 2–3 — Consultant Version (Background Duties)

**Question 2.** Should the patient be extubated today? Please select one option and briefly justify your decision.

- Yes, I extubate the patient.
- No, I wait and reassess first thing tomorrow morning.

On a scale of 1–5, how confident are you in this decision?

**Question 3.** Should antibiotic therapy be discontinued today?

- Yes, I discontinue antibiotic therapy.
- No, I wait and reassess first thing tomorrow morning.

On a scale of 1–5, how confident are you in this decision?

**Decision Questions 2–3 — Resident Version (Foreground Duties)**

**Question 2.** Should the patient be extubated today? *(Identical question and options to the consultant version above.)* On a scale of 1–5, how confident are you in this decision?

**Question 3.** Unfortunately, the arterial line has become dislodged during nursing care. The pulse oximetry waveform remains good. Do you insert a new arterial line? Please select one option and briefly justify your decision.

- Yes, I insert a new arterial line for the patient.
- No, I wait and reassess the situation tomorrow.

On a scale of 1–5, how confident are you in this decision?

## S2.5 — Scenario 5: Patient with Basal Ganglia Haemorrhage

**Patient Profile**

- Male, 72 years, 110 kg, 182 cm (BMI 33.2 $kg/m^2$)
- **Diagnosis:**
  - Status post left basal ganglia haemorrhage with intraventricular extension, 14 days ago
  - Aspiration pneumonia (MSSA in tracheal aspirate, 3 days after admission)
- **Past medical history:**
  - Arterial hypertension
  - Paroxysmal atrial fibrillation, previously on anticoagulation with a direct oral anticoagulant (DOAC)
  - Chronic kidney disease, dialysis-dependent, left arm arteriovenous fistula
  - Peripheral arterial disease, Fontaine stage III–IV
  - Status post aorto-bifemoral bypass (8 years ago)
  - Status post multiple bilateral femoropopliteal bypasses, most recently revised 3 years ago
  - Status post thrombendarterectomy of both femoral arteries
  - Multiple percutaneous transluminal angioplasty/stent interventions of the iliac and femoral arteries

**Current Status**

The patient was extubated 2 days ago and remains mildly compromised pulmonarily. Adequate oxygenation is achieved with high-flow nasal cannula (HFNC) and intermittent non-invasive ventilation. Reduced alertness, but awake and responsive. Marked right-sided hemiparesis, central facial palsy, expressive aphasia. External ventricular drain still in situ. No new neurological deficits. Circulation stable and catecholamine-free; known atrial fibrillation. Antibiotic therapy was discontinued one day ago (total duration 10 days). Over recent days, core temperature has remained <38.0 °C, but has been mildly elevated for the past 6 hours despite metamizole and paracetamol (most recently 38.3 °C). Intermittent (Monday–Wednesday–Friday) GENIUS dialysis via Shaldon catheter.

Central venous catheter and Shaldon catheter (right internal jugular vein), arterial line (left radial artery), and nasogastric tube were placed on the day of admission and have not been exchanged since. The central venous catheter insertion site is mildly erythematous.

**Cerebrospinal Fluid Findings**

| Parameter | Finding | Reference range |
|---|---|---|
| Colour/appearance | Xanthochromic, mildly turbid | Clear, colourless |
| Erythrocytes (/$\mu$L) | 25,000 | 0 |
| Leukocytes (/$\mu$L) | 35 | $< 5$ |
| Segmented granulocytes (%) | 30 | $< 10$ |
| Lymphocytes (%) | 55 | 60–90 |
| Monocytes/macrophages (%) | 15 | $< 10$ |
| Protein (mg/dL) | 160 | $< 45$ |
| Glucose (mg/dL) | 60 | 50–75 ($\geq$2/3 serum) |
| Lactate (mmol/L) | 2.2 | $< 2.4$ |
| Microscopy/Gram stain | No organisms identified | – |
| Culture | No growth after 48 h | – |
| PCR (if performed) | Negative | – |

**Arterial Blood Gas Series (Current Day)**

| Parameter | 08:00 | 12:00 | 16:00 | 20:00 |
|---|---|---|---|---|
| pH | 7.47 | 7.45 | 7.43 | 7.41 |
| $PaO_2$ (mmHg) | 72 | 70 | 68 | 66 |
| $PaCO_2$ (mmHg) | 32 | 34 | 35 | 36 |
| $HCO_3^-$ (mmol/L) | 23 | 23 | 23 | 22 |
| Base excess (mmol/L) | +1 | 0 | 0 | −1 |
| $SaO_2$ (%) | 92 | 91 | 90 | 89 |
| Hb (g/dL) | 8.6 | 8.4/8.6[a] | 8.2/8.5[a] | 7.9/8.4[a] |
| Lactate (mg/dL) | 14 | 16 | 18 | 19/22[a] |
| $Na^+$ (mmol/L) | 147/141[a] | 146/141[a] | 148/142[a] | 147/142[a] |
| $K^+$ (mmol/L) | 4.6 | 4.7 | 4.9 | 5.1 |
| $iCa^{2+}$ (mmol/L) | 1.08 | 1.08 | 1.07 | 1.07 |
| $Cl^-$ (mmol/L) | 110 | 111 | 112 | 112 |
| Glucose (mg/dL) | 185 | 180 | 175 | 170 |

**[a]Values differed between the two role-specific scenario versions: Hb and lactate at 20:00 and sodium across all timepoints (consultant version / resident version).**

**Laboratory Parameters (Days 12–14)**

| Parameter | Day 12 | Day 13 | Day 14 |
|---|---|---|---|
| Hb (g/dL)[a] | 7.9/9.3 | 8.5/8.8 | 7.7/8.4 |
| Hct (%) | 28 | 26 | 25 |
| Platelets (/nL) | 270 | 230 | 200 |
| Leukocytes (/nL) | 10.8 | 11.2 | 14.5 |
| CRP (mg/L) | 50 | 70 | 110 |
| Procalcitonin (ng/mL) | 0.7 | 1.2 | 2.1 |
| INR | 1.3 | 1.4 | 1.4 |
| aPTT (s) | 30 | 31 | 33 |
| Creatinine (mg/dL) | 3.5 | 2.7 | 3.9 |
| Urea (mg/dL) | 110 | 70 | 101 |
| Bilirubin (mg/dL) | 1.6 | 1.6 | 1.5 |

**[a]Haemoglobin values differed between the two role-specific scenario versions (consultant version / resident version) for all three days.**

**Decision Questions — Consultant Version (Background Duties)**

**Question 1.** Should the central venous catheter and Shaldon catheter be exchanged today? Please select one option and briefly justify your decision.

- Yes, the lines need to be exchanged today.
- No, I wait and reassess first thing tomorrow morning.

On a scale of 1–5, how confident are you in this decision (1 = very uncertain, 5 = very confident)?

**Question 2.** Should a fresh red cell concentrate be transfused? Please select one option and briefly justify your decision.

- Yes, the patient requires transfusion of a red cell concentrate.
- No, I wait and reassess first thing tomorrow morning.

On a scale of 1–5, how confident are you in this decision?

**Question 3.** The arterial line has not been giving a reliable reading for one to two hours. The responsible nurse asks whether you would like a new line placed. Please select one option and briefly justify your decision.

- Yes, the patient requires an arterial line; I insert a new one.
- No, I have the malfunctioning line removed and do not insert a new one.

On a scale of 1–5, how confident are you in this decision?

**Decision Questions — Resident Version (Foreground Duties)**

**Question 1.** Should the central venous catheter and Shaldon catheter be exchanged today? *(Identical question and options to the consultant version above.)* On a scale of 1–5, how confident are you in this decision?

**Question 2.** Should antibiotic therapy be resumed? Please select one option and briefly justify your decision.

- Yes, I restart antibiotic therapy with piperacillin/tazobactam.
- No, I wait and reassess first thing tomorrow morning.

On a scale of 1–5, how confident are you in this decision?

**Question 3.** Should a chest X-ray be performed today? Please select one option and briefly justify your decision.

- Yes, I arrange a chest X-ray today.
- No, I wait and reassess first thing tomorrow morning.

On a scale of 1–5, how confident are you in this decision?

## S2.6 — Scenario 6: Neurosurgical Patient with Pneumonia

Scenarios 6 and 7 describe the same patient followed across two consecutive observation days; Scenario 6 covers Days 1–4, and Scenario 7 (Section S2.7) continues from Day 5. As in the preceding scenarios, two role-specific sets of three decision questions were administered (consultant/background and resident/foreground; see Table S3).

### Patient Profile

- Male, 39 years, 184 cm, 98 kg (BMI 29 $kg/m^2$)
- **Current diagnoses:**
  - WHO grade II astrocytoma, right hemisphere (status post craniotomy and tumour resection 8 days ago)
  - Large space-occupying postoperative haemorrhage on Day 2 (re-operation: right hemicraniectomy, haematoma evacuation)
  - Bilateral pneumonia (ventilator-associated) since Day 4
  - Harmful alcohol use and nicotine use (20 pack-years)

### Clinical Course, Days 1–2

**Day 1 — ICU admission.** Postoperative admission following right-sided craniotomy for astrocytoma. The patient is haemodynamically stable, postoperatively intubated and ventilated. Due to the long duration of surgery and a restless first night on the ICU, extubation is deferred to the following day. Pupillary checks were consistently unremarkable. Dexamethasone 4 mg three times daily for oedema prophylaxis.

**Day 2.** The patient did not wake adequately postoperatively, showing only undirected arousal with sporadic eye opening and non-specific withdrawal to pain. No purposeful motor responses, no command following. Pupils isocoric and reactive. A left-sided hemiparesis was noted, with reduced spontaneous movement and absent withdrawal to pain on that side.

**Cranial CT:** A large intracerebral haemorrhage in the right hemisphere, arising from the fronto-temporal resection cavity. Extensive hyperdense haemorrhage extending into the right ventricular system, with compression of the right lateral ventricle and midline shift of approximately 9 mm to the left. Associated perifocal oedema, with tendency towards subfalcine herniation. No signs of acute ischaemia. Skull bone shows the postoperative defect from the right craniotomy, with air collections in the operative bed. **Summary:** extensive postoperative rebleeding in the resection cavity with midline shift.

The patient underwent emergency revision surgery with right decompressive craniectomy, haematoma evacuation, and placement of an external ventricular drain (EVD). He was returned to the ICU postoperatively ventilated and under analgosedation, haemodynamically stable on low-dose noradrenaline.

**Follow-up cranial CT after emergency surgery:** Status post right decompressive craniectomy with largely decompressed operative field. Residual hyperdense blood products within the former resection cavity. Midline shift reduced to approximately 3 mm, lateral ventricles now of normal configuration. Perifocal hypodense oedema persists in the right temporo-parietal region. The external ventricular drain, via the right frontal horn, is in correct position; the ventricular system is slim, with small residual intraventricular blood. No new haemorrhage or acute ischaemia. Air in the operative bed is decreasing.

### Clinical Course, Days 3–4

**Day 3.** Still under analgosedation, circulation stable. Pupils bilaterally mid-sized and reactive. No evidence of epileptic activity. The EVD drains adequate volumes of bloody cerebrospinal fluid. ICP remains stable at low-normal values. Sedation is being slowly reduced in preparation for a spontaneous awakening trial the following day.

**Day 4.** Due to worsening respiratory function, with rising ventilator pressures and increased secretion burden, the awakening trial is deferred. Microbiological samples are obtained (tracheal aspirate, blood cultures). Antibiotic therapy with ceftriaxone is started (no risk factors for multidrug-resistant organisms, no shock). Urine output has become more restricted over the course of the day (40–50 mL/h).

*Additional information (Resident version):* Current fluid balance is +1200 mL.

**Chest X-ray:** New bilateral patchy, partly confluent infiltrates, more pronounced basally and on the right than the left. Diaphragmatic borders blunted bilaterally, with reduced dorsobasal transparency. Heart size normal, central vascular markings prominent. No free air under the diaphragm, no pleural effusions clearly identifiable. Endotracheal tube tip correctly positioned 3 cm above the carina; nasogastric tube and central venous catheter in correct position.

| Parameter | Day 1 | Day 2 | Day 3 | Day 4 |
|---|---|---|---|---|
| Hb (g/dL) | 10.1 | 9.6 | 8.8 | 8.2 |
| Hct (%) | 31 | 29 | 27 | 25 |
| Platelets (/nL) | 290 | 260 | 230 | 190 |
| Leukocytes (/nL) | 11.8 | 13.2 | 14.8 | 19.5 |
| CRP (mg/L) | 28 | 65 | 140 | 260 |
| Procalcitonin (ng/mL) | 0.4 | 0.9 | 1.2 | 3.5 |
| INR | 1.2 | 1.2 | 1.4 | 1.3 |
| aPTT (s) | 28 | 29 | 31 | 33 |
| Creatinine (mg/dL) | 1.2 | 1.6 | 2.2 | 2.9 |
| Urea (mg/dL) | 52 | 64 | 82 | 98 |
| Bilirubin (mg/dL) | 1.0 | 1.2 | 1.3 | 1.2 |

| Parameter | 12:00 | 14:00 | 16:00 |
|---|---|---|---|
| pH | 7.33 | 7.29 | 7.25 |
| $PaO_2$ (mmHg) | 74 | 68 | 72 |
| $PaCO_2$ (mmHg) | 47 | 52 | 56 |
| $HCO_3^-$ (mmol/L) | 24 | 23 | 22 |
| Base excess (mmol/L) | −2 | −3 | −4 |
| $SaO_2$ (%) | 93 | 90 | 87 |
| Lactate (mg/dL) | 11 | 13 | 15 |
| $Na^+$ (mmol/L) | 141 | 142 | 142 |
| $K^+$ (mmol/L) | 4.0 | 4.1 | 4.2 |
| $Cl^-$ (mmol/L) | 109 | 110 | 111 |
| Glucose (mg/dL) | 175 | 182 | 188 |
| *Ventilation (BiVent mode)* | | | |
| $FiO_2$ (%) | 50 | 55 | 60 |
| PEEP (mbar) | 10 | 11 | 12 |
| Peak pressure (mbar) | 28 | 30 | 31 |
| Plateau pressure (mbar) | 25 | 26 | 27 |
| Tidal volume (mL) | 490 | 480 | 470 |
| Respiratory rate (/min) | 18 | 20 | 22 |
| Minute ventilation (L/min) | 8.8 | 9.6 | 10.3 |
| *Monitoring* | | | |
| Heart rate (/min) | 92 | 98 | 105 |
| BP (mmHg) | 118/68 (85) | 112/66 (81) | 96/54 (68) |
| $SpO_2$ (%) | 92 | 90 | 88 |
| ICP (mmHg) | 10 | 12 | 13 |
| Noradrenaline (mg/h) | 0.4 | 0.6 | 0.8 |

**Laboratory Trend, Days 1–4**

**Arterial Blood Gas, Ventilator and Monitoring Parameters, Day 4**

**Decision Questions — Consultant Version (Background Duties)**

**Question 1.** Should the patient receive continuous furosemide therapy via syringe driver? Please select one option and briefly justify your decision.

- Yes, I start a furosemide infusion.
- No, I wait and reassess the situation the following day.

On a scale of 1–5, how confident are you in this decision (1 = very uncertain, 5 = very confident)?

**Question 2.** Should the patient have a chest CT? Please select one option and briefly justify your decision.

- Yes, I order the CT examination.
- No, I wait and reassess the situation the following day.

On a scale of 1–5, how confident are you in this decision?

**Question 3.** Should the patient be transfused with a red cell concentrate?

- Yes, I transfuse a red cell concentrate.
- No, I wait and reassess the situation the following day.

On a scale of 1–5, how confident are you in this decision?

**Decision Questions — Resident Version (Foreground Duties)**

**Question 1.** Should the patient receive a furosemide bolus? Please select one option and briefly justify your decision.

- Yes, I administer a furosemide bolus.
- No, I wait and reassess the situation the following day.

On a scale of 1–5, how confident are you in this decision?

**Question 2.** Should the patient have a chest CT? *(Identical question and options to the consultant version above.)* On a scale of 1–5, how confident are you in this decision?

**Question 3.** Should the patient be transfused with a red cell concentrate? *(Identical question and options to the consultant version above.)* On a scale of 1–5, how confident are you in this decision?

## S2.7 — Scenario 7: Neurosurgical Patient with Pneumonia (Continuation)

Scenario 7 continues directly from Scenario 6 (Section S2.6), describing the same patient's course from Day 5.

### Clinical Course, Day 5

Under ongoing therapy (negative fluid balance, ceftriaxone), the respiratory situation continues to deteriorate.

**Plain chest CT:** Extensive, partly confluent bilateral ground-glass opacities and consolidations, more pronounced basally and dorsobasally. Relatively better-aerated areas ventrally. Marked dystelectasis in the dependent lung regions. Small bilateral pleural effusions. No abscess formation, no pneumothorax. Heart size and central vessels unremarkable. The patient is scheduled for prone positioning later that evening.

| Parameter | Day 5 |
|---|---|
| Hb (g/dL) | 7.9 |
| Hct (%) | 24 |
| Platelets (/nL) | 180 |
| Leukocytes (/nL) | 27.0 |
| CRP (mg/L) | 310 |
| Procalcitonin (ng/mL) | 4.2 |
| INR | 1.4 |
| aPTT (s) | 31 |
| Creatinine (mg/dL) | 3.1 |
| Urea (mg/dL) | 101 |

**Day 5 laboratory parameters. In the resident version, serum albumin was additionally reported (26.1 g/L), consistent with that version's decision question regarding albumin administration.**

### Ventilator, Monitoring and Arterial Blood Gas Parameters, Day 5 (12:00–16:00)

| Parameter | 12:00 | 14:00 | 16:00 |
|---|---|---|---|
| *Ventilation (BiVent mode)* | | | |
| $FiO_2$ (%) | 70 | 75 | 80 |
| PEEP (mbar) | 12 | 12 | 14 |
| Peak pressure (mbar) | 30 | 32 | 32 |
| Plateau pressure (mbar) | 30 | 33 | 33 |
| Tidal volume (mL) | 440 | 420 | 400 |
| Respiratory rate (/min) | 22 | 22 | 22 |
| Minute ventilation (L/min) | 10.4 | 9.6 | 8.9 |
| *Monitoring* | | | |
| Heart rate (/min) | 102 | 103 | 105 |
| BP (mmHg) | 95/58 (67) | 90/50 (63) | 89/49 (62) |
| $SpO_2$ (%) | 92 | 93 | 93 |
| ICP (mmHg) | 13 | 12 | 13 |
| Noradrenaline (mg/h) | 0.8 | 1.1 | 1.3 |

| Parameter | 12:00 | 16:00 |
|---|---|---|
| pH | 7.27 | 7.25 |
| $PaO_2$ (mmHg) | 68 | 65 |
| $PaCO_2$ (mmHg) | 56 | 58 |
| $HCO_3^-$ (mmol/L) | 22 | 21 |
| Base excess (mmol/L) | −4 | −5 |
| $SaO_2$ (%) | 88 | 86 |
| Hb (g/dL) | 7.6 | 7.5 |
| Lactate (mg/dL) | 15 | 16 |
| $Na^+$ (mmol/L) | 142 | 142 |
| $K^+$ (mmol/L) | 4.2 | 4.3 |
| $iCa^{2+}$ (mmol/L) | 1.06 | 1.05 |
| $Cl^-$ (mmol/L) | 111 | 112 |
| Glucose (mg/dL) | 188 | 190 |

**Day 5 arterial blood gas series, supine position.**

**Decision Question 1 — Consultant Version (Background Duties)**

**Question 1.** Do you escalate antibiotic therapy to meropenem ± vancomycin? Please select one option and briefly justify your decision.

- Yes, I escalate therapy.
- No, I wait and reassess the situation the following day.

On a scale of 1–5, how confident are you in this decision (1 = very uncertain, 5 = very confident)?

**Decision Question 1 — Resident Version (Foreground Duties)**

**Question 1.** Should the patient receive a fluid bolus of 200 g human albumin? Please select one option and briefly justify your decision.

- Yes, I administer the fluid bolus.
- No, I wait and reassess the situation the following day.

On a scale of 1–5, how confident are you in this decision?

**Course After Prone Positioning (Both Versions)**

After one hour in the prone position, the patient suddenly develops atrial fibrillation (maximum heart rate 170/min) with acute haemodynamic deterioration. Catecholamine requirements increase. Rate control to 135/min is achieved with beta-blockers and magnesium.

**Decision Questions 2–3 (Both Versions)**

**Question 2.** Do you reverse the prone position? Please select one option and briefly justify your decision.

- Yes, I return the patient to the supine position.
- No, I wait and reassess the situation later.

On a scale of 1–5, how confident are you in this decision?

| Parameter | 17:00 | 18:00 | 19:00 |
|---|---|---|---|
| pH | 7.33 | 7.36 | 7.29 |
| $PaO_2$ (mmHg) | 92 | 98 | 65 |
| $PaCO_2$ (mmHg) | 48 | 46 | 49 |
| $HCO_3^-$ (mmol/L) | 24 | 25 | 25 |
| Base excess (mmol/L) | −2 | −1 | −3 |
| $SaO_2$ (%) | 95 | 97 | 93 |
| Hb (g/dL) | 7.4 | 7.4 | 7.3 |
| Lactate (mg/dL) | 12 | 11 | 15 |
| $Na^+$ (mmol/L) | 141 | 141 | 141 |
| $K^+$ (mmol/L) | 4.0 | 4.0 | 4.0 |
| $iCa^{2+}$ (mmol/L) | 1.08 | 1.09 | 1.08 |
| $Cl^-$ (mmol/L) | 109 | 108 | 107 |
| Glucose (mg/dL) | 175 | 170 | 172 |
| *Ventilation (BiVent mode)* | | | |
| $FiO_2$ (%) | 80 | 70 | 70 |
| PEEP (mbar) | 14 | 14 | 14 |
| Peak pressure (mbar) | 32 | 32 | 32 |
| Plateau pressure (mbar) | 33 | 33 | 33 |
| Tidal volume (mL) | 400 | 440 | 420 |
| Respiratory rate (/min) | 22 | 22 | 22 |
| Minute ventilation (L/min) | 8.9 | 10.8 | 9.9 |
| *Monitoring* | | | |
| Heart rate (/min) | 102 | 103 | 135 |
| BP (mmHg) | 95/58 (67) | 90/50 (63) | 89/49 (62) |
| $SpO_2$ (%) | 95 | 96 | 93 |
| ICP (mmHg) | 13 | 12 | 13 |
| Noradrenaline (mg/h) | 0.5 | 0.7 | 1.3 |

**Arterial blood gas, ventilator, and monitoring parameters following prone positioning, Day 5.**

**Question 3.** Do you arrange a chest CT to rule out pulmonary embolism?

- Yes, I obtain the CT pulmonary angiogram.
- No, I wait and reassess the situation later.

On a scale of 1–5, how confident are you in this decision?

## S2.8 — Scenario 8: Patient following a Whipple Procedure

As in the preceding scenarios, two role-specific sets of three decision questions were administered (consultant/background and resident/foreground; see Table S3). Both versions share the same patient profile, current status, and monitoring/laboratory data; only Decision Question 1 is identical across versions, whereas Questions 2 and 3 differ between the consultant and resident versions.

### Patient Profile

- Male, 79 years, 182 cm, 95 kg (BMI 29 $kg/m^2$), privately insured
- **Current diagnoses:**
  - Cholangiocarcinoma
  - Status post Whipple procedure with left-sided hemihepatectomy (2 days ago)
  - Pre-obesity (overweight, WHO classification)
  - Known two-vessel coronary artery disease
  - Permanent atrial fibrillation
  - Arterial hypertension
  - Insulin-dependent diabetes mellitus
  - Moderate nicotine and alcohol use

### Current Medication

| Medication | Dose / route |
|---|---|
| Heparin, continuous i.v. | 1200 IU/h (target aPTT 50–60 s) |
| Metoprolol 5 mg p.o. | 1-0-1 |
| Digoxin 0.125 mg p.o. | 1-0-0 |
| Aspirin 100 mg p.o. | 1-0-0 |
| Atorvastatin 40 mg p.o. | 1-0-0 |
| Ramipril 2.5 mg p.o. | 1-0-0 |
| Pantoprazole 40 mg p.o. | 1-0-0 |
| Metamizole drops p.o., 1 g | 40-40-40-40 |
| Insulin s.c., sliding scale | target-value oriented |

**Current Status**

The patient has been on the surgical ICU for 48 hours postoperatively. The first 24 hours were characterised by mild catecholamine dependency (noradrenaline 0.1–0.3 mg/h); since this morning the patient has been catecholamine-free, stable, and alert. He is well analgised with a thoracic epidural catheter in place. Overnight, the patient received a single 10 mg furosemide bolus from the on-call physician because of low hourly urine output. $SpO_2$ is 96% on 2 L/min $O_2$ via nasal cannula. He has already been mobilised to the edge of the bed and tolerated a light breakfast (tea, soup, porridge). The patient is expected to be transferred to the general ward following the surgical ward round.

**Vital Signs**

| Parameter | Value |
|---|---|
| Heart rate | 95 /min (atrial fibrillation) |
| Blood pressure | 105/55 mmHg |
| $SpO_2$ | 96% on 2 L $O_2$/min (93% on room air) |
| Temperature | 37.4 °C |
| Respiratory rate | 15 /min |
| Urine output | 40 mL/h |

**ECG:** A 12-lead ECG was provided, showing the underlying permanent atrial fibrillation with a ventricular rate of approximately 95 /min (not reproduced here).

**Arterial Blood Gas, Day 2**

| Parameter | 08:00 | 10:00 |
|---|---|---|
| pH | 7.47 | 7.45 |
| $PaO_2$ (mmHg) | 89 | 88 |
| $PaCO_2$ (mmHg) | 36 | 39 |
| $HCO_3^-$ (mmol/L) | 23 | 23 |
| Base excess (mmol/L) | +1 | 0 |
| $SaO_2$ (%) | 95 | 96 |
| Hb (g/dL) | 8.0 | 7.9 |
| Lactate (mg/dL) | 6 | 8 |
| $Na^+$ (mmol/L) | 141 | 141 |
| $K^+$ (mmol/L) | 4.1 | 4.0 |
| $iCa^{2+}$ (mmol/L) | 1.08 | 1.08 |
| $Cl^-$ (mmol/L) | 110 | 111 |
| Glucose (mg/dL) | 112 | 144 |

**Laboratory Trend, Days 1–2**

**Drain fluid lipase, Day 2:** 45 U/L.

**Decision Question 1 (Both Versions)**

**Question 1.** Should the patient be transfused with a red cell concentrate? Please select one option and briefly justify your decision.

- Yes, I transfuse a red cell concentrate.

| Parameter | Day 1 | Day 2 |
|---|---|---|
| Hb (g/dL) | 8.8 | 7.4 |
| Hct (%) | 26 | 21 |
| Platelets (/nL) | 330 | 290 |
| Leukocytes (/nL) | 11.2 | 9.5 |
| CRP (mg/L) | 70 | 78 |
| Procalcitonin (ng/mL) | 0.11 | 0.12 |
| INR | 1.4 | 1.4 |
| aPTT (s) | 31 | 54 |
| Creatinine (mg/dL) | 1.1 | 1.4 |
| Urea (mg/dL) | 50 | 65 |
| Bilirubin (mg/dL) | 1.6 | 1.5 |
| AST/GOT (U/L) | 85 | 68 |
| ALT/GPT (U/L) | 72 | 72 |
| GGT (U/L) | 240 | 210 |
| ALP (U/L) | 170 | 155 |

- No, I arrange regular Hb monitoring, documented in the discharge letter.

On a scale of 1–5, how confident are you in this decision (1 = very uncertain, 5 = very confident)?

**Decision Questions 2–3 — Consultant Version (Background Duties)**

**Question 2.** Should the patient receive a loop diuretic (furosemide, torasemide) as standing medication? Please select one option and briefly justify your decision.

- Yes, I prescribe an additional loop diuretic for the patient.
- No, I do not prescribe an additional loop diuretic and note in the discharge letter that urine output should be monitored regularly.

On a scale of 1–5, how confident are you in this decision?

**Question 3.** Following the general surgical ward round, the patient is due to be transferred in order to free the bed for an urgent admission. Do you transfer the patient to the general ward?

- Yes, I transfer the patient to the general ward.
- No, the patient should be monitored for a further night (e.g. because of the urine output and Hb trend).

On a scale of 1–5, how confident are you in this decision?

**Decision Questions 2–3 — Resident Version (Foreground Duties)**

**Question 2.** The nurse draws your attention to the potassium value on the arterial blood gas and asks whether you would like to start a potassium infusion (via syringe driver). Please select one option and briefly justify your decision.

- Yes, I prescribe a potassium infusion.
- No, I will wait for the next measurement.

On a scale of 1–5, how confident are you in this decision?

**Question 3.** The nurse draws your attention to the patient's sparse urine output. Should the patient receive a further furosemide bolus?

- Yes, I prescribe a furosemide bolus.
- No, I will wait a little longer to observe the trend in urine output.

On a scale of 1–5, how confident are you in this decision?

# 3 Supplement S3 — Recruitment and Observer Details

## Participant Recruitment

Participants were recruited from intensivists working in the Department of Anaesthesiology of a German University Hospital operating three surgical intensive care units. The study was conducted on the general surgical ICU. Recruitment was carried out through direct personal approach by the principal investigator (A.L.L.) on the unit. No financial or material incentives were provided.

The consultant sample comprised all three consultants routinely assigned to the general surgical ICU. As the unit operates with a fixed consultant body, all eligible consultants were approached and all agreed to participate. This recruitment approach reflects the practical constraints of a small, fixed clinical team rather than a purposive sampling strategy, and as such introduces the possibility of volunteer bias.

Residents were approached selectively based on their level of ICU experience, with the aim of capturing a range of clinical seniority within the resident sample. Specifically, residents in their first or second ICU rotation were prioritised to complement the experienced consultant sample. All residents approached agreed to participate. One resident (a04) was subsequently excluded from all analyses owing to an unplanned absence that precluded the completion of a sufficient number of observed shifts.

All participants received written and oral information about the study from the research team prior to enrolment, and written informed consent was obtained before the first observation shift and before the publication of pseudonymised data.

## Observer Training and Positionality

Shadowing was carried out by a single trained observer (F.H.), a senior medical student with prior professional experience as an ICU nurse. F.H. had no supervisory or hierarchical relationship with any of the participating physicians. Prior to the observation period, F.H. received structured training from the research team covering the decision classification system, the real-time data collection protocol, and the use of the digital tally counter application. A one-week pilot phase was conducted in February 2025 to finalise these procedures (see Methods). F.H. took no part in patient care during any observation shift.

The use of a single observer throughout the study ensured consistency of data collection but also means that inter-rater reliability could not be assessed. The potential for observer influence on physician behaviour (Hawthorne effect) cannot be excluded, though the repeated presence of the observer across multiple shifts was intended to minimise this risk by allowing participants to habituate to being observed.

## End-of-Life Decisions

End-of-life decisions were defined as decisions to withhold or withdraw life-sustaining therapy (e.g. vasopressors, mechanical ventilation, renal replacement therapy) or to transition a patient's goals of care towards comfort-focused management. These decisions were recorded as a distinct subcategory within the tally counter but, given their low frequency ($n = 7$ across 38 shifts), were retained in the overall decision count and not analysed separately.

## Sample Size Considerations

Given the exploratory nature of this study and the absence of comparable prior data on burstiness as a predictor of psychometric outcomes in ICU physicians, no formal a priori power calculation was performed. The sample of five physicians and 38 shifts was determined by feasibility constraints — specifically, the availability of a single trained observer (F.H.) and the fixed consultant body of the unit (see above). Post hoc, the high intraclass correlation coefficients (Supplement S7) indicate that the study was adequately powered to detect between-physician differences but underpowered for shift-level effects of smaller magnitude (see Limitations).

# 4 Supplement S4 — Sensitivity and Robustness Analyses

## Overview

Primary analyses used evening psychometric scores (the decision-fatigue, sleepiness, and mood scores) as outcome variables, reflecting the cumulative state of each physician at the end of the shift. The robustness of the inferentially reported associations — a higher mean decision rate with a higher evening decision-fatigue score, a higher burstiness with a lower evening mood score, and the adjusted contrasts between shift types for the evening mood score — was examined with several complementary approaches: (i) an alternative outcome specification using daily mean scores; (ii) bounded-influence robust estimation; (iii) a rank transformation of the outcome; (iv) parametric bootstrap confidence intervals for the two key workload coefficients; and (v), for the single-item sleepiness score, a cumulative-link mixed model acknowledging its ordinal nature. All models retained physician as a random intercept and, where applicable, were fitted by maximum likelihood, identical to the primary specification. The outcome distributions and the residual diagnostics of the primary models are reported in Supplement S8.

## Workload Models (Daily Mean Scores)

Results from the sensitivity analysis using daily mean scores were broadly consistent with the primary analyses. A higher mean decision rate remained positively associated with mean decision-fatigue score ($b = 2.87$, $SE = 1.12$, $p = 0.018$), confirming that the association between decision volume and fatigue was not confined to the end of the shift. Burstiness remained negatively associated with mean mood score ($b = -48.3$, $SE = 14.1$, $p = 0.003$), consistent with the primary finding. No other workload variable reached statistical significance in either the primary or sensitivity analyses.

## Shift-Type Models (Daily Mean Scores)

Associations between shift type and daily mean mood score were consistent with the primary analyses. All three non-reference shift types showed significantly lower daily mean mood disturbance than quiet-smooth shifts in unadjusted models (quiet-clustered: $b = -10.8$, $p < 0.001$; busy-smooth: $b = -7.4$, $p = 0.002$; busy-clustered: $b = -9.3$, $p < 0.001$), and these associations remained robust after adjustment (all $p \leq 0.005$). Associations between shift type and daily mean decision-fatigue and sleepiness scores were not statistically significant, consistent with the primary analyses.

## Robust Mixed-Effects Models

To assess the influence of outlying shifts on the fixed-effect estimates, all primary models were refitted using bounded-influence robust estimation (`robustlmm`), which downweights observations and clusters with large residuals rather than excluding them. This method does not provide $p$-values by design; robust point estimates and standard errors are reported. The two workload associations retained their direction with attenuated magnitude relative to the primary models (mean decision rate on the decision-fatigue score: $b = 3.80$, $SE = 1.44$, cf. 4.13; burstiness on the mood score: $b = -49.62$, $SE = 17.54$, cf. $-62.61$). The adjusted shift-type contrasts for the mood score remained substantial under robust estimation (quiet-clustered $b = -9.94$; busy-smooth $b = -7.80$; busy-clustered $b = -7.37$; each versus quiet-smooth).

## Rank-Transformed Models

As a distribution-free check, each outcome was replaced by its rank across the 38 shifts and the same mixed-effects models were refitted; the resulting coefficients are on the rank scale and test for a monotone rather than a linear association. The burstiness–mood association retained significance ($b = -40.88$, $SE = 18.58$, $p = 0.035$), whereas the decision-rate–fatigue association was reduced to a non-significant trend ($b = 5.22$, $SE = 2.69$, $p = 0.061$). In the adjusted shift-type model, only the quiet-clustered versus quiet-smooth contrast remained significant after rank transformation ($b = -5.97$, $SE = 2.21$, $p = 0.011$), whereas the busy-smooth and busy-clustered contrasts were markedly attenuated and non-significant ($b = -0.30$, $p = 0.903$ and $b = -0.30$, $p = 0.911$, respectively). This indicates that the raw-scale mood advantage of the busy shift types over quiet-smooth shifts is partly driven by a small number of quiet-smooth shifts with extreme mood-disturbance values (predominantly those of the least experienced consultant; see main text) and should be interpreted accordingly. The decision severity index, by contrast, remained strongly associated with a lower mood score on the rank scale ($b = -94.66$, $p < 0.001$), consistent with the primary model.

## Parametric Bootstrap Confidence Intervals

Confidence intervals for the two primary workload coefficients were additionally obtained by parametric bootstrap (`lme4::confint`, `method = "boot"`, 5000 simulations, fixed seed). A minority of resamples produced boundary (singular) fits or convergence warnings, which is expected given the small number of random-effect levels (five physicians) and does not invalidate the percentile intervals obtained from the converged replicates. The bootstrap interval for the mean decision rate on the decision-fatigue score was $[1.18,\ 7.14]$ and that for burstiness on the mood score was $[-101.95,\ -22.99]$; both excluded zero, consistent with the Satterthwaite-based inference.

## Ordinal Model for the Sleepiness Score

Because the evening sleepiness score is a single ordinal nine-point item rather than a continuous scale, a cumulative-link mixed model (`ordinal::clmm`) with a physician random intercept and $z$-standardised predictors was additionally attempted. This model did not fit reliably: although the Hessian condition number ($3.0 \times 10^5$) remained below the conventional $1 \times 10^6$ instability threshold, the standard errors of all fixed effects collapsed to near-identical values of approximately 0.002, producing implausibly large coefficient-to-standard-error ratios (all $|z| > 45$) and effectively degenerate inference. The ordinal model was therefore judged numerically unreliable, and its estimates are not reported or interpreted. Because the primary linear model showed no significant workload association with the sleepiness score and its residuals were unremarkable (Supplement S8), the linear specification is retained for this outcome, with the acknowledgement that a single ordinal item is not ideally treated as continuous.

## Interpretation

Across specifications, the two primary workload associations retained their direction — positive for the mean decision rate on the decision-fatigue score, negative for burstiness on the mood score. The burstiness–mood association was the most robust, surviving the daily-mean, robust, rank-transformed, and bootstrap specifications; the decision-rate–fatigue association was directionally stable but weakened to a trend under the rank transformation. The adjusted shift-type contrasts for the mood score were robust for the quiet-clustered

Table S4: Robustness of the key fixed-effect estimates across model specifications. Values are estimates ($b$) with standard errors in parentheses. Primary = linear mixed-effects model with Satterthwaite $p$; Robust = bounded-influence estimation (no $p$ by design); Rank = rank-transformed outcome. Shift-type contrasts are versus quiet-smooth from the adjusted model. All models include physician as a random intercept; $n = 38$ shifts.

| Effect | Primary $b$ (SE), $p$ | Robust $b$ (SE) | Rank $b$ (SE), $p$ |
|---|---|---|---|
| Decision rate → fatigue | 4.13 (1.50), 0.010 | 3.80 (1.44) | 5.22 (2.69), 0.061 |
| Burstiness → mood | −62.61 (20.30), 0.004 | −49.62 (17.54) | −40.88 (18.58), 0.035 |
| quiet-clustered → mood (adj.) | −10.46 (2.52), <0.001 | −9.94 (1.67) | −5.97 (2.21), 0.011 |
| busy-smooth → mood (adj.) | −9.62 (2.80), 0.002 | −7.80 (1.85) | −0.30 (2.46), 0.903 |
| busy-clustered → mood (adj.) | −9.95 (3.03), 0.002 | −7.37 (2.01) | −0.30 (2.67), 0.911 |

comparison but sensitive to the rank transformation for the busy-smooth and busy-clustered comparisons, reflecting the influence of a few extreme quiet-smooth shifts and the small sample; the association between a higher decision severity index and a lower mood score was likewise robust across specifications. The cumulative-link mixed model for the ordinal sleepiness score did not fit reliably and was not interpreted. The primary analyses using evening scores are retained as the principal specification, subject to the caveats noted here.

## 5 Supplement S5 — Shift-Type Descriptive Statistics

Table S5 presents descriptive statistics for all shift-level metrics and psychometric outcomes separately by shift type. Values represent means with standard deviations in parentheses. Shift types were defined by median split of decision density (mean decision rate per 5-minute interval; median = 3.27) and temporal clustering (burstiness; median = 0.38), yielding four groups: quiet-smooth ($n = 5$), quiet-clustered ($n = 14$), busy-smooth ($n = 14$), and busy-clustered ($n = 5$).

Table S5: Descriptive statistics by shift type. Values are means ($SD$). QS = quiet-smooth; QC = quiet-clustered; BS = busy-smooth; BC = busy-clustered. DR = mean decision rate (decisions per 5-minute interval); SI = decision severity index; $B$ = burstiness; Fatigue, Sleepiness, Mood = evening decision-fatigue, sleepiness, and mood scores (see Methods); $p$(cons.) = proportion of conservative scenario responses; Cert. = mean response certainty (1–5 scale).

| Metric | QS ($n = 5$) | QC ($n = 14$) | BS ($n = 14$) | BC ($n = 5$) |
|---|---|---|---|---|
| Total decisions | 447 (46) | 427 (32) | 517 (35) | 499 (24) |
| DR | 3.08 (0.31) | 2.96 (0.22) | 3.58 (0.24) | 3.46 (0.17) |
| $B$ | 0.328 (0.031) | 0.444 (0.039) | 0.333 (0.028) | 0.397 (0.012) |
| SI | 1.091 (0.038) | 1.091 (0.044) | 1.109 (0.041) | 1.123 (0.060) |
| Interruptions | 34.4 (3.4) | 20.4 (5.0) | 28.4 (9.1) | 27.4 (9.4) |
| Breaks ($n$) | 2.4 (0.5) | 2.1 (0.8) | 2.8 (0.7) | 2.8 (0.4) |
| Break duration (min) | 76.0 (34.2) | 71.4 (28.7) | 83.9 (36.3) | 80.0 (21.5) |
| Fatigue (evening) | 22.2 (10.6) | 14.8 (7.6) | 18.5 (7.0) | 18.2 (7.6) |
| Sleepiness (evening) | 4.8 (2.2) | 3.2 (1.7) | 3.8 (1.6) | 4.0 (1.0) |
| Mood (evening) | +22.6 (23.0) | −5.8 (12.3) | −0.4 (12.1) | +0.8 (12.1) |
| $p$(cons.) | 0.40 (0.28) | 0.45 (0.28) | 0.48 (0.25) | 0.60 (0.15) |
| Cert. | 4.13 (0.51) | 4.14 (0.50) | 3.86 (0.47) | 3.80 (0.56) |

Table S6 reports the corresponding mixed-effects model estimates for the association between shift type and evening psychometric scores, both unadjusted and adjusted for total decision count, decision severity index, interruptions, breaks, and break duration.

| | | Fatigue | | Sleepiness | | Mood | |
|---|---|---|---|---|---|---|---|
| Predictor | Model | $b$ (SE) | $p$ | $b$ (SE) | $p$ | $b$ (SE) | $p$ |
| quiet-clustered | Unadj. | −0.51 (1.41) | 0.722 | −0.36 (0.55) | 0.514 | **−13.0** (2.39) | **< 0.001** |
| | Adj. | −0.66 (1.48) | 0.656 | −0.51 (0.58) | 0.385 | **−10.5** (2.52) | **< 0.001** |
| busy-smooth | Unadj. | +2.32 (1.44) | 0.117 | +0.05 (0.56) | 0.933 | **−9.06** (2.44) | **0.001** |
| | Adj. | +0.38 (1.64) | 0.821 | +0.30 (0.64) | 0.648 | **−9.60** (2.81) | **0.002** |
| busy-clustered | Unadj. | +1.21 (1.71) | 0.483 | −0.08 (0.67) | 0.900 | **−11.2** (2.89) | **< 0.001** |
| | Adj. | +0.14 (1.78) | 0.940 | +0.24 (0.70) | 0.734 | **−9.95** (3.04) | **0.002** |
| *Significant covariates (adjusted models):* | | | | | | | |
| dec_total | Adj. | **+0.03** (0.01) | **0.046** | +0.00 (0.01) | 0.975 | +0.03 (0.02) | 0.277 |
| decision severity index | Adj. | −24.3 (15.4) | 0.124 | **−11.9** (5.84) | **0.048** | **−76.1** (26.3) | **0.007** |

Table S6: Associations between shift type and evening psychometric scores. Fatigue, Sleepiness, and Mood denote the evening decision-fatigue, sleepiness, and mood scores (see Methods). Bold = $p < 0.05$; $n = 38$ shifts, physician as random intercept.

# 6 Supplement S6 — Scenario Model: Full Results

Table S7 presents the full results of the binomial mixed-effects models for conservative scenario responses and the linear mixed-effects model for response certainty by shift type. All comparisons were non-significant; findings are strictly exploratory given the small number of scenarios per shift ($k = 3$) and the limited sample size.

| | OR unadjusted | | OR adjusted | | Certainty |
|---|---|---|---|---|---|
| Shift type | OR (95 % CI) | $p$ | OR (95 % CI) | $p$ | $b$ (SE) |
| quiet-smooth | Ref. | | Ref. | | Ref. |
| quiet-clustered | 1.24 [0.37, 4.11] | 0.949 | 1.33 [0.31, 5.64] | 0.939 | −0.47 (0.28) |
| busy-smooth | 1.36 [0.41, 4.52] | 0.892 | 2.88 [0.55, 15.14] | 0.451 | −0.48 (0.24) |
| busy-clustered | 2.25 [0.52, 9.70] | 0.555 | 4.31 [0.72, 25.94] | 0.264 | −0.58 (0.29) |

All OR comparisons: $p > 0.26$. All certainty shift-type contrasts: $p > 0.05$.
Interruptions → certainty: $b = -0.029$, $SE = 0.011$, $p = 0.012$.

Table S7: Shift type and scenario conservatism and response certainty (reference: quiet-smooth). ORs with 95 % CIs from binomial mixed-effects models (unadjusted and adjusted); certainty estimates from a linear mixed-effects model with interruptions and evening decision-fatigue score as covariates. All comparisons non-significant; findings are strictly exploratory.

# 7 Supplement S7 — Random Effects, ICC, and Trajectory Model Outputs

## Random Effects and Intraclass Correlation Coefficients

Table S8 presents the between-physician variance components and intraclass correlation coefficients (ICC) for all linear mixed-effects models. ICC was calculated as $\hat{\sigma}^2_{\text{physician}}/(\hat{\sigma}^2_{\text{physician}} + \hat{\sigma}^2_{\text{residual}})$, where $\hat{\sigma}^2_{\text{physician}}$ reflects stable between-physician differences in psychometric baseline levels and $\hat{\sigma}^2_{\text{residual}}$ reflects shift-to-shift variability within physicians. High ICCs confirm that the majority of total variance was attributable to between-physician differences, justifying the use of random intercepts throughout. The near-zero ICC for response certainty is consistent with the singular fit observed in that model and indicates negligible systematic differences between physicians in scenario certainty ratings.

Table S8: Random-effect variance components and ICC by model. Var(Physician) = between-physician variance; Var(Residual) = within-physician residual variance; ICC = intraclass correlation coefficient. Primary models used evening psychometric scores as outcomes; sensitivity models used daily mean scores. Fatigue, Sleepiness, Mood = decision-fatigue, sleepiness, and mood scores; Certainty = scenario response certainty.

| Outcome | Model | Var(Physician) | Var(Residual) | ICC |
|---|---|---|---|---|
| *Primary workload models (evening scores)* | | | | |
| Fatigue | Workload predictors | 74.59 | 6.30 | 0.922 |
| Sleepiness | Workload predictors | 2.22 | 0.99 | 0.692 |
| Mood | Workload predictors | 224.80 | 27.91 | 0.890 |
| *Sensitivity models (daily mean scores)* | | | | |
| Fatigue | Workload predictors | 55.52 | 2.88 | 0.951 |
| Sleepiness | Workload predictors | 1.13 | 0.30 | 0.790 |
| Mood | Workload predictors | 208.32 | 16.05 | 0.928 |
| *Shift-type models — unadjusted (evening scores)* | | | | |
| Fatigue | Shift type | 62.62 | 3.55 | 0.946 |
| Sleepiness | Shift type | 1.47 | 0.46 | 0.761 |
| Mood | Shift type | 243.95 | 20.19 | 0.924 |
| *Shift-type models — adjusted (evening scores)* | | | | |
| Fatigue | Shift type + covariates | 68.29 | 3.70 | 0.949 |
| Sleepiness | Shift type + covariates | 1.83 | 0.38 | 0.827 |
| Mood | Shift type + covariates | 243.41 | 21.31 | 0.919 |
| *Scenario model* | | | | |
| Certainty | Shift type + interruptions | 0.01 | 0.19 | 0.050 |

The near-zero ICC for response certainty is consistent with the singular fit observed in this model and reflects negligible between-physician differences in response certainty.

## Trajectory Models

To examine whether psychometric scores changed across the shift and whether the trajectory differed by shift type, linear mixed-effects models were fitted with timepoint (morning, noon, evening) as a within-shift repeated measure, shift type as a between-shift predictor, and their interaction. Morning scores served as the baseline ($\Delta = 0$); the intercept therefore reflects the estimated mean for the quiet-smooth group at morning. Because treatment contrasts were used, the timepoint terms describe within-shift change in the quiet-smooth reference group only and must not be read as the average change across all shift types; interaction terms indicate whether the trajectory of that change differed between a given shift type and quiet-smooth. Change

averaged across all shifts is reported separately below. All models included physician as a random intercept. Tables S9, S10, and S11 present the full fixed-effect outputs for the mood, decision-fatigue, and sleepiness scores respectively.

Table S9: Trajectory model: mood score. Linear mixed-effects model with timepoint × shift-type interaction; quiet-smooth = reference; morning = baseline ($\Delta = 0$). Physician as random intercept. $n = 38$ shifts, 5 physicians.

| Term | $b$ | $SE$ | $df$ | $p$ |
|---|---|---|---|---|
| (Intercept) | 1.56 | 7.40 | 6.98 | 0.840 |
| Timepoint: Noon | 7.95 | 3.33 | 62.22 | 0.020 |
| Timepoint: Evening | 8.49 | 4.25 | 20.14 | 0.059 |
| Shift: quiet-clustered | -3.01 | 3.61 | 39.94 | 0.410 |
| Shift: busy-smooth | 1.70 | 3.67 | 40.00 | 0.645 |
| Shift: busy-clustered | -1.74 | 4.36 | 39.93 | 0.693 |
| Noon × quiet-clustered | −11.97 | 3.57 | 75.29 | 0.001 |
| Evening × quiet-clustered | −9.69 | 3.90 | 55.01 | 0.016 |
| Noon × busy-smooth | −11.87 | 3.59 | 75.59 | 0.001 |
| Evening × busy-smooth | −10.48 | 3.97 | 55.54 | 0.011 |
| Noon × busy-clustered | −9.26 | 4.33 | 75.21 | 0.036 |
| Evening × busy-clustered | −9.33 | 4.71 | 54.86 | 0.053 |

Table S10: Trajectory model: decision-fatigue score. Linear mixed-effects model with timepoint × shift-type interaction; quiet-smooth = reference; morning = baseline ($\Delta = 0$). Physician as random intercept. $n = 38$ shifts, 5 physicians.

| Term | $b$ | $SE$ | $df$ | $p$ |
|---|---|---|---|---|
| (Intercept) | 15.45 | 3.41 | 6.75 | 0.003 |
| Timepoint: Noon | 2.03 | 1.62 | 78.00 | 0.214 |
| Timepoint: Evening | 0.66 | 1.73 | 38.83 | 0.703 |
| Shift: quiet-clustered | -0.31 | 1.58 | 46.95 | 0.844 |
| Shift: busy-smooth | 3.46 | 1.60 | 47.28 | 0.035 |
| Shift: busy-clustered | -0.49 | 1.91 | 46.82 | 0.798 |
| Noon × quiet-clustered | -2.84 | 1.87 | 76.39 | 0.133 |
| Evening × quiet-clustered | 0.07 | 1.94 | 63.50 | 0.970 |
| Noon × busy-smooth | -4.60 | 1.87 | 77.06 | 0.016 |
| Evening × busy-smooth | -0.65 | 1.96 | 62.02 | 0.740 |
| Noon × busy-clustered | -1.41 | 2.27 | 76.13 | 0.537 |
| Evening × busy-clustered | 2.18 | 2.35 | 63.74 | 0.357 |

Table S11: Trajectory model: sleepiness score. Linear mixed-effects model with timepoint × shift-type interaction; quiet-smooth = reference; morning = baseline ($\Delta = 0$). Physician as random intercept. $n = 38$ shifts, 5 physicians.

| Term | $b$ | $SE$ | $df$ | $p$ |
|---|---|---|---|---|
| (Intercept) | 3.60 | 0.58 | 19.02 | <0.001 |
| Timepoint: Noon | 0.40 | 0.45 | 62.30 | 0.382 |
| Timepoint: Evening | 0.20 | 0.67 | 27.71 | 0.766 |
| Shift: quiet-clustered | -0.10 | 0.54 | 41.56 | 0.856 |
| Shift: busy-smooth | 0.11 | 0.54 | 41.63 | 0.835 |
| Shift: busy-clustered | -0.46 | 0.65 | 41.39 | 0.480 |
| Noon × quiet-clustered | -0.96 | 0.51 | 75.92 | 0.063 |
| Evening × quiet-clustered | -0.10 | 0.71 | 37.92 | 0.888 |
| Noon × busy-smooth | -0.85 | 0.51 | 75.55 | 0.097 |
| Evening × busy-smooth | 0.11 | 0.71 | 37.55 | 0.883 |
| Noon × busy-clustered | -0.64 | 0.62 | 75.85 | 0.303 |
| Evening × busy-clustered | 0.52 | 0.86 | 37.85 | 0.547 |

## Within-Shift Change Averaged Across Shift Types

Because the timepoint terms of the models above refer to the quiet-smooth reference group, within-shift change for the sample as a whole was estimated separately using models without the shift-type interaction (timepoint as the only fixed effect; random intercepts and time slopes for physicians and for shifts nested within physicians; for the decision-fatigue score this structure was singular, and random intercepts only were used). Contrasts against the morning baseline are shown in Table S12, together with descriptive means.

Averaged across all 38 shifts, none of the three scores showed a statistically significant change from morning to evening, and all confidence intervals included zero. This is consistent with the interaction models: the increase in mood disturbance observed in quiet-smooth shifts was offset by the absence of such an increase in the remaining shift types, so that the average trajectory across the sample was essentially flat. Within-shift change therefore differed by shift type rather than occurring uniformly across the cohort.

Table S12: Within-shift change relative to the morning baseline, averaged across all shift types. Estimates are from linear mixed-effects models with timepoint as the only fixed effect ($n$ = 38 shifts, 5 physicians). Descriptive means with standard deviations are given for reference.

| Score | Contrast | $b$ | $SE$ | 95 % CI | | $p$ |
|---|---|---|---|---|---|---|
| Decision-fatigue score | Noon − morning | −0.90 | 0.64 | −2.34 | 0.55 | 0.286 |
| | Evening − morning | +0.74 | 0.64 | −0.71 | 2.18 | 0.415 |
| Sleepiness score | Noon − morning | −0.35 | 0.24 | −0.95 | 0.25 | 0.288 |
| | Evening − morning | +0.28 | 0.41 | −0.90 | 1.45 | 0.736 |
| Mood score | Noon − morning | −2.06 | 1.82 | −6.52 | 2.40 | 0.444 |
| | Evening − morning | −0.17 | 2.97 | −8.69 | 8.34 | 0.995 |
| *Descriptive means (SD)* | | | | | | |
| Decision-fatigue score | morning 16.8 (7.9); noon 15.9 (7.9); evening 17.6 (7.9) | | | | | |
| Sleepiness score | morning 3.50 (1.27); noon 3.13 (1.53); evening 3.74 (1.66) | | | | | |
| Mood score | morning 1.3 (16.7); noon −0.9 (18.4); evening 0.8 (16.1) | | | | | |

# 8 Supplement S8 — Model Diagnostics and Unmodelled Temporal Covariates

## Outcome Distributions and Descriptive Statistics

Table S13 summarises the distribution of each psychometric outcome at the three daily timepoints, and Figure S1 shows the corresponding histograms and kernel-density estimates. All three outcomes were positively (right) skewed at every timepoint (skewness 0.68–1.64) and departed significantly from normality on the Shapiro–Wilk test (all $p \leq 0.001$). The mood score was the most dispersed and most skewed, reflecting a subset of shifts — predominantly those of the least experienced consultant — with pronounced mood disturbance. Because the psychometric scores were analysed with linear mixed-effects models, the relevant distributional assumption concerns the model residuals rather than the raw outcomes; the Shapiro–Wilk tests on raw values additionally ignore the clustering within physicians and are of limited power at $n = 38$, and are therefore reported descriptively only. Residual diagnostics are reported below, and the sensitivity of the inferential conclusions to these distributional features is examined in Supplement S4.

Table S13: Distributional characteristics of the psychometric outcomes by timepoint ($n = 38$ shifts). IQR = interquartile range $[Q_{25}, Q_{75}]$; Skew. = skewness; Kurt. = kurtosis; $W$, $p$ = Shapiro–Wilk statistic and $p$-value. Shapiro–Wilk tests were computed on raw shift-level values, ignore clustering within physicians, and are reported descriptively only.

| Score | Timepoint | Mean (SD) | Median [IQR] | Range | Skew. | Kurt. | $W$ | $p$ |
|---|---|---|---|---|---|---|---|---|
| Decision-fatigue | Morning | 16.84 (7.93) | 13.0 [10.3, 22.3] | 10 to 37 | 0.95 | 2.62 | 0.818 | <0.001 |
| | Noon | 15.95 (7.92) | 12.5 [10.3, 14.8] | 10 to 34 | 1.30 | 3.12 | 0.720 | <0.001 |
| | Evening | 17.58 (7.88) | 14.0 [12.0, 21.8] | 10 to 34 | 0.93 | 2.52 | 0.834 | <0.001 |
| Sleepiness | Morning | 3.50 (1.27) | 3.0 [3.0, 4.0] | 2 to 6 | 0.68 | 2.40 | 0.856 | <0.001 |
| | Noon | 3.13 (1.53) | 3.0 [2.0, 4.0] | 1 to 7 | 0.70 | 2.60 | 0.892 | 0.001 |
| | Evening | 3.74 (1.66) | 3.0 [2.0, 5.0] | 2 to 7 | 0.75 | 2.53 | 0.855 | <0.001 |
| Mood | Morning | 1.29 (16.71) | −5.5 [−9.8, 4.0] | −15 to 41 | 1.27 | 3.19 | 0.777 | <0.001 |
| | Noon | −0.92 (18.38) | −7.0 [−14.0, −2.3] | −16 to 53 | 1.64 | 4.57 | 0.740 | <0.001 |
| | Evening | 0.82 (16.11) | −4.5 [−9.8, 1.8] | −15 to 44 | 1.37 | 3.76 | 0.803 | <0.001 |

## Residual Diagnostics

Model assumptions were checked by inspection of residuals-versus-fitted plots and quantile–quantile plots of both the residuals and the estimated physician random intercepts, for the primary workload models (decision-fatigue, sleepiness, and mood scores) and the adjusted shift-type model for the mood score (Figure S2). Despite the non-normality of the raw outcomes noted above, the model residuals showed no substantial deviation from normality or homoscedasticity, and the random-intercept quantile–quantile plots (five physicians) revealed no gross departures. For the two models exhibiting singular fits (response certainty; conservative scenario responses), the near-zero between-physician variance is reported in Table S8, and results from these models should be interpreted with appropriate caution.

## Weekday and Shift Sequence Position

Weekday and the position of a shift within a physician's consecutive working sequence were not included as covariates. Whilst circadian and cumulative fatigue effects across consecutive shifts are plausible, the present

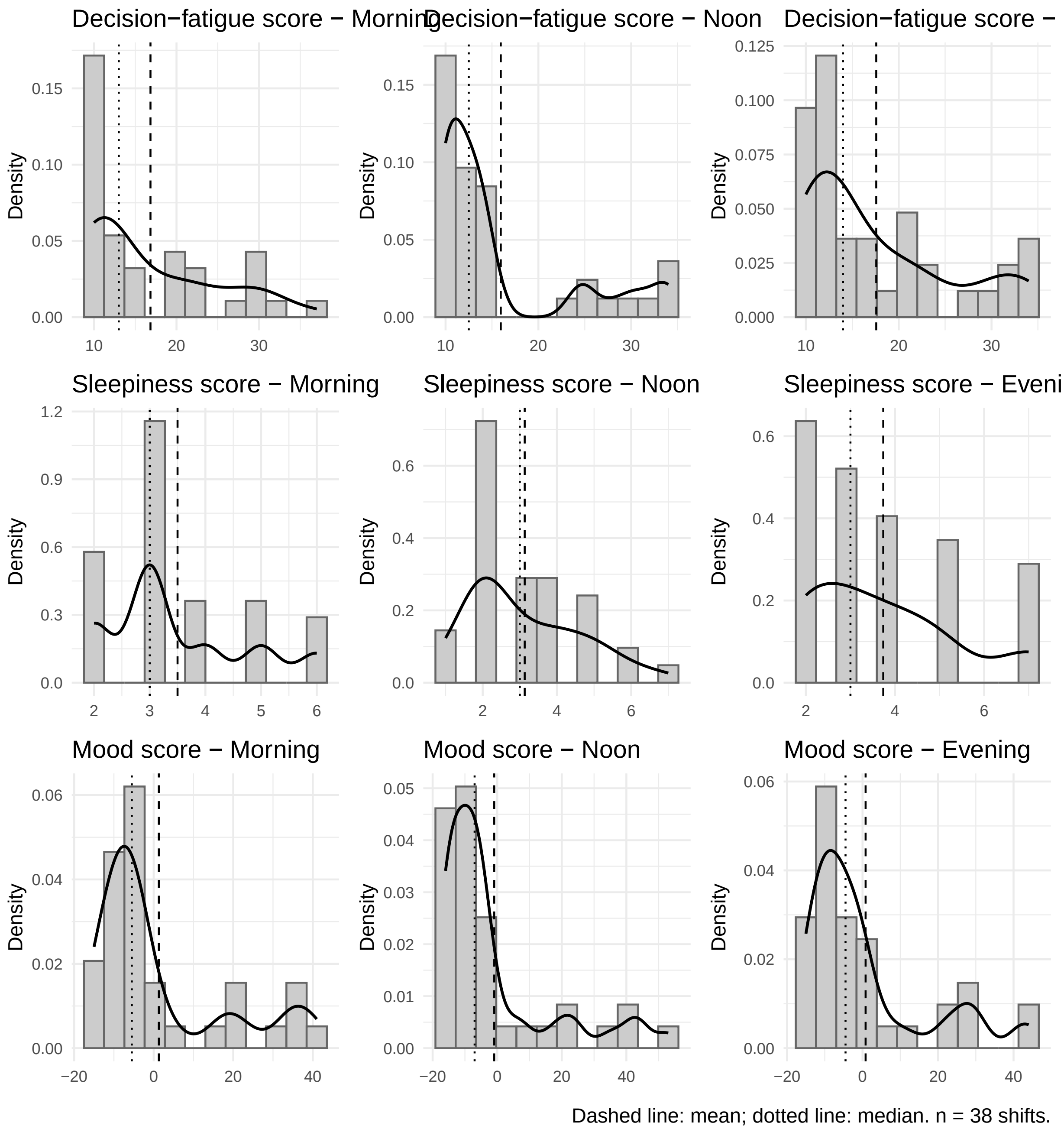


Figure S1: Distribution of the three psychometric outcomes (decision-fatigue, sleepiness, and mood scores) at the morning, noon, and evening timepoints. Bars show histograms (density scale); solid curves show kernel-density estimates. Dashed line = mean; dotted line = median. $n = 38$ shifts.

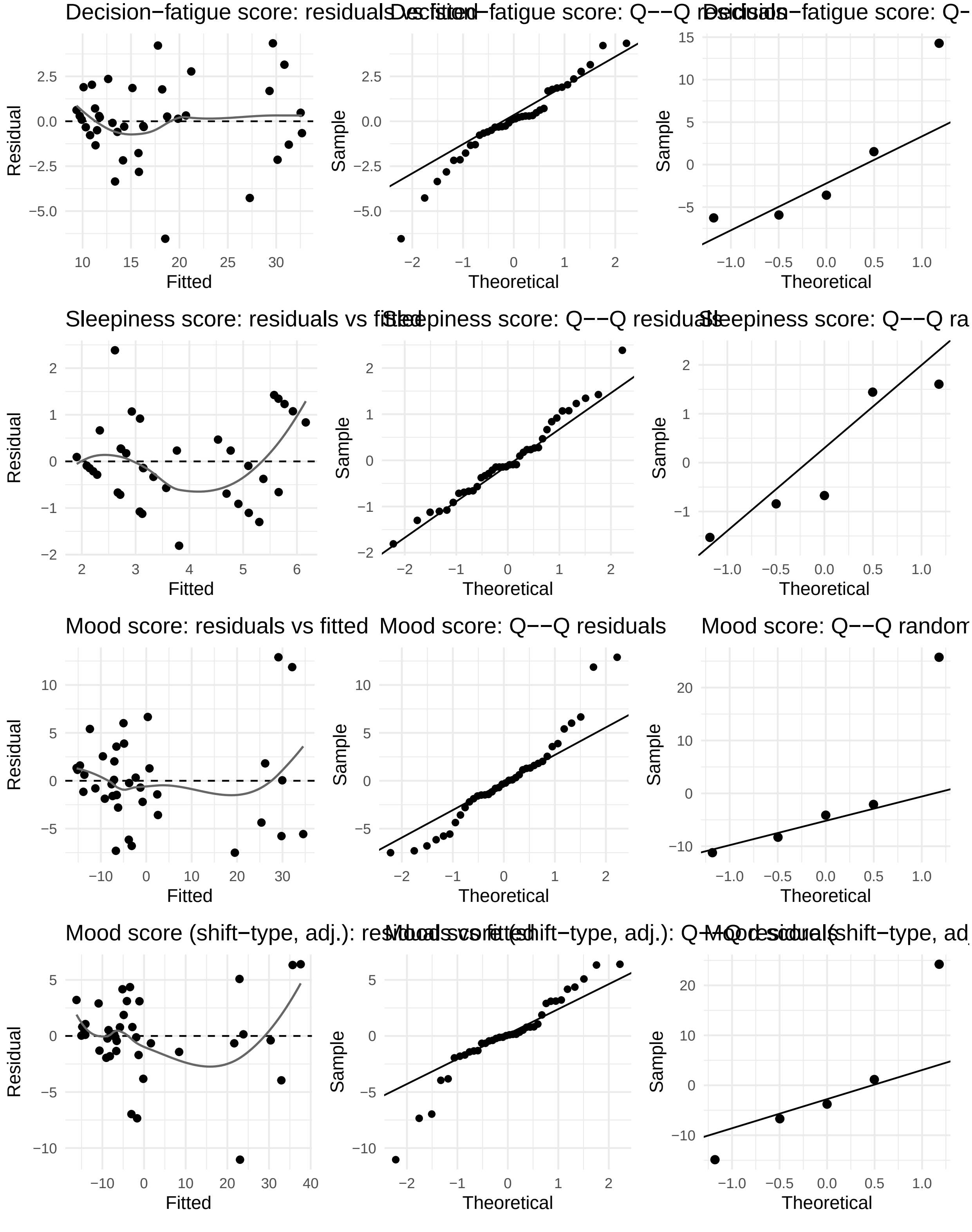


Figure S2: Residual diagnostics for the primary linear mixed-effects models. Rows (top to bottom): decision-fatigue score, sleepiness score, and mood score (each with workload predictors), and the mood score under the adjusted shift-type model. Columns (left to right): residuals versus fitted values, quantile–quantile plot of the residuals, and quantile–quantile plot of the estimated physician random intercepts ($n = 5$).

sample did not permit reliable estimation of such effects given the limited number of shifts per physician and the resulting collinearity with the physician random intercept. This is noted as a direction for future research with larger samples (see Discussion).

# 9 Supplement S9 — Additional Data Files

The following anonymised data files are provided as additional supplementary material and can be used to reproduce the workload metrics, shift-type classification, and psychometric analyses reported in the main text and in Supplements S4–S8.

Table S14: Overview of additional supplementary data files. All files are provided in comma-separated value (CSV) format with one header row. Physician identifiers correspond to those used in Table ?? of the main manuscript.

| File name | Content |
|---|---|
| `master_clean.csv` | Shift-level dataset ($n = 38$ rows), one row per observed shift. Variables include physician identifier, day number, decision counts by severity (`dec_small`, `dec_medium`, `dec_large`, `dec_eol`), number of interruptions, morning/noon/evening decision-fatigue, sleepiness, and mood scores, the binary decision and response certainty (1–5) for each of the three scenario questions, the scenario identifier, derived conservative/active ("safe"/"unsafe") classification variables and proportions, and the number and total duration of breaks. |
| `bins_5min_clean.csv` | Item-level time-series dataset ($n = 5472$ rows; 38 shifts × 144 five-minute bins, corresponding to 12-hour shifts), containing the physician identifier, day number, shift date, bin start timestamp, and the number of recorded decision events within each five-minute bin. This file underlies the calculation of the mean decision rate and burstiness reported in `workload_metrics.csv` and in Supplement S5. |
| `workload_metrics.csv` | Shift-level dataset ($n = 38$ rows), derived from `bins_5min_clean.csv`, containing the mean decision rate (`mean_rate`; decisions per five-minute bin), the standard deviation of the bin-count series (`sd_rate`), and the burstiness parameter (`burstiness`), calculated as $B = (CV - 1)/(CV + 1)$ with $CV = SD/\text{mean}$ of the 144 bin counts per shift. |

The decision severity index (SI) reported in Supplement S5 was calculated from `master_clean.csv` by assigning ordinal weights to each decision category (small = 1, medium = 2, large = 3) and computing their mean across all small, medium, and large decisions recorded during a shift (end-of-life decisions were not included in this calculation), thereby quantifying the average clinical complexity of the shift's decision load. The shift-type classification (quiet-smooth, quiet-clustered, busy-smooth, busy-clustered; Supplement S5) was obtained by a median split of the mean decision rate and burstiness values in `workload_metrics.csv`.

Free-text justifications accompanying the three binary decision questions per scenario were recorded as part of the original data collection but were judged not relevant to the quantitative analyses reported here and were therefore not included in this anonymised data deposit and not analysed further.

These files are deposited alongside this manuscript and are available from the corresponding author upon reasonable request, subject to the data protection constraints described in the main text (Declarations).

# 10 Supplement S10 — Per-Shift Data Listing

Table S15 lists the complete set of shift-level metrics and evening psychometric scores for each of the 38 observed shifts, corresponding to the deposited file `master_clean.csv` (Supplement S9). Each row represents one twelve-hour shift.

| | | | Decisions | | | | | Temporal structure | | | Interruptions & breaks | | | Scenarios | | Evening scores | | |
|---|---|---|---|---|---|---|---|---|---|---|---|---|---|---|---|---|---|---|
| Physician | Day | Shift | Small | Med | Large | EoL | Total | DR | SI | Burst | Int | Brk | Min | CD | Cert | Fatigue | Sleep. | Mood |
| a01 | 1 | QC | 377 | 35 | 0 | 0 | 412 | 2.86 | 1.08 | 0.48 | 17 | 2 | 90 | 0.33 | 4.00 | 22 | 5 | −7 |
| a01 | 2 | BS | 415 | 76 | 0 | 0 | 491 | 3.41 | 1.15 | 0.37 | 14 | 3 | 100 | 0.67 | 3.67 | 24 | 3 | −9 |
| a01 | 3 | QC | 384 | 71 | 0 | 0 | 455 | 3.16 | 1.16 | 0.43 | 17 | 2 | 50 | 0.33 | 4.33 | 12 | 2 | −12 |
| a01 | 4 | BS | 423 | 81 | 0 | 0 | 504 | 3.50 | 1.16 | 0.33 | 19 | 2 | 40 | 0.33 | 4.00 | 20 | 2 | −8 |
| a01 | 5 | BS | 426 | 55 | 0 | 0 | 481 | 3.34 | 1.11 | 0.37 | 13 | 3 | 75 | 0.67 | 5.00 | 21 | 4 | −5 |
| a01 | 6 | BC | 411 | 70 | 0 | 0 | 481 | 3.34 | 1.15 | 0.40 | 29 | 2 | 60 | 0.67 | 3.67 | 20 | 3 | −7 |
| a01 | 7 | BS | 413 | 63 | 0 | 0 | 476 | 3.31 | 1.13 | 0.36 | 26 | 2 | 75 | 0.67 | 3.67 | 19 | 4 | −7 |
| a01 | 8 | QC | 311 | 76 | 0 | 0 | 387 | 2.68 | 1.20 | 0.41 | 28 | 2 | 75 | 0.67 | 4.00 | 13 | 2 | −14 |
| a02 | 1 | BS | 469 | 61 | 0 | 0 | 530 | 3.68 | 1.12 | 0.30 | 24 | 3 | 95 | 0.33 | 4.67 | 13 | 7 | 1 |
| a02 | 2 | BC | 439 | 88 | 0 | 0 | 527 | 3.66 | 1.17 | 0.38 | 19 | 3 | 65 | 0.33 | 4.67 | 10 | 5 | −10 |
| a02 | 3 | QC | 359 | 39 | 0 | 0 | 398 | 2.76 | 1.10 | 0.41 | 22 | 4 | 140 | 0.33 | 3.33 | 10 | 5 | 2 |
| a02 | 4 | BS | 408 | 91 | 1 | 1 | 501 | 3.47 | 1.19 | 0.29 | 23 | 2 | 50 | 0.33 | 4.00 | 12 | 7 | −1 |
| a02 | 5 | QS | 413 | 74 | 0 | 0 | 487 | 3.26 | 1.15 | 0.37 | 35 | 3 | 130 | 0.33 | 4.67 | 13 | 4 | 7 |
| a02 | 6 | QC | 396 | 65 | 0 | 0 | 461 | 3.20 | 1.14 | 0.45 | 24 | 1 | 75 | 1.00 | 5.00 | 12 | 5 | −3 |
| a03 | 1 | BS | 448 | 31 | 0 | 0 | 479 | 3.33 | 1.06 | 0.35 | 39 | 3 | 70 | 0.33 | 3.67 | 15 | 3 | −3 |
| a03 | 2 | QC | 437 | 29 | 1 | 1 | 468 | 3.24 | 1.07 | 0.42 | 19 | 2 | 90 | 0.33 | 4.33 | 12 | 2 | −8 |
| a03 | 3 | QC | 389 | 40 | 0 | 0 | 429 | 2.98 | 1.09 | 0.40 | 26 | 2 | 45 | 0.33 | 3.67 | 12 | 3 | −10 |
| a03 | 4 | BS | 549 | 40 | 0 | 0 | 589 | 4.09 | 1.07 | 0.32 | 35 | 2 | 60 | 1.00 | 3.67 | 14 | 3 | −4 |
| a03 | 5 | BC | 448 | 25 | 0 | 0 | 473 | 3.28 | 1.05 | 0.41 | 22 | 3 | 80 | 0.67 | 3.33 | 14 | 3 | 1 |
| a03 | 6 | BS | 473 | 27 | 1 | 1 | 502 | 3.47 | 1.06 | 0.38 | 28 | 4 | 170 | 0.33 | 3.33 | 16 | 3 | −2 |
| a03 | 7 | BC | 459 | 33 | 0 | 0 | 492 | 3.41 | 1.07 | 0.40 | 24 | 3 | 115 | 0.67 | 4.00 | 17 | 4 | −1 |
| a03 | 8 | BS | 513 | 52 | 2 | 1 | 568 | 3.93 | 1.10 | 0.31 | 32 | 3 | 90 | 0.00 | 3.67 | 16 | 3 | −2 |
| a05 | 1 | BS | 476 | 34 | 0 | 0 | 510 | 3.52 | 1.07 | 0.33 | 29 | 3 | 125 | 0.33 | 3.67 | 13 | 3 | −8 |
| a05 | 2 | BS | 512 | 38 | 1 | 0 | 551 | 3.77 | 1.07 | 0.32 | 38 | 2 | 40 | 0.67 | 3.67 | 11 | 2 | −11 |
| a05 | 3 | QS | 440 | 27 | 0 | 0 | 467 | 3.24 | 1.06 | 0.31 | 31 | 2 | 55 | 0.00 | 4.67 | 10 | 2 | −9 |
| a05 | 4 | QC | 422 | 31 | 1 | 1 | 455 | 3.15 | 1.07 | 0.44 | 21 | 3 | 70 | 0.67 | 4.33 | 12 | 2 | −13 |
| a05 | 5 | QC | 408 | 29 | 0 | 0 | 437 | 3.03 | 1.07 | 0.45 | 15 | 2 | 90 | 0.00 | 3.67 | 10 | 2 | −13 |
| a05 | 6 | QC | 393 | 20 | 0 | 0 | 413 | 2.87 | 1.05 | 0.48 | 17 | 2 | 60 | 0.67 | 4.67 | 10 | 2 | −14 |
| a05 | 7 | QC | 433 | 27 | 0 | 0 | 460 | 3.18 | 1.06 | 0.44 | 24 | 1 | 30 | 0.33 | 4.00 | 10 | 2 | −14 |
| a05 | 8 | QC | 402 | 35 | 0 | 0 | 437 | 3.03 | 1.08 | 0.44 | 27 | 2 | 45 | 0.00 | 4.00 | 10 | 2 | −15 |
| a06 | 1 | BS | 442 | 74 | 0 | 1 | 517 | 3.58 | 1.14 | 0.32 | 38 | 3 | 120 | 0.33 | 3.33 | 33 | 4 | 24 |
| a06 | 2 | BC | 424 | 96 | 0 | 0 | 520 | 3.61 | 1.18 | 0.39 | 43 | 3 | 80 | 0.67 | 3.33 | 30 | 5 | 21 |
| a06 | 3 | QS | 424 | 43 | 1 | 1 | 469 | 3.25 | 1.10 | 0.35 | 31 | 3 | 55 | 0.33 | 4.00 | 34 | 7 | 42 |
| a06 | 4 | QC | 375 | 27 | 0 | 0 | 402 | 2.79 | 1.07 | 0.41 | 18 | 2 | 45 | 0.67 | 3.67 | 34 | 7 | 28 |
| a06 | 5 | BS | 493 | 51 | 0 | 0 | 544 | 3.77 | 1.09 | 0.31 | 40 | 4 | 65 | 0.67 | 4.00 | 32 | 5 | 30 |
| a06 | 6 | QC | 349 | 17 | 0 | 0 | 366 | 2.54 | 1.05 | 0.55 | 11 | 3 | 95 | 0.67 | 5.00 | 28 | 4 | 12 |
| a06 | 7 | QS | 335 | 34 | 0 | 0 | 369 | 2.53 | 1.09 | 0.31 | 39 | 2 | 90 | 0.67 | 3.67 | 23 | 4 | 29 |
| a06 | 8 | QS | 419 | 26 | 0 | 0 | 445 | 3.09 | 1.06 | 0.30 | 36 | 2 | 50 | 0.67 | 3.67 | 31 | 7 | 44 |

Table S15: Descriptive statistics ($n = 38$). DR = mean decision rate per 5-min interval; SI = decision severity index; Burst = burstiness; Int = interruptions; Brk = breaks; Min = total break duration (min); CD = proportion of conservative scenario responses; Cert = mean certainty (1–5); Fatigue, Sleep., Mood = evening decision-fatigue, sleepiness, and mood scores; EoL = end-of-life decisions. QS/QC/BS/BC = quiet-smooth/quiet-clustered/busy-smooth/busy-clustered.

# References

[1] Eirik H. Ofstad et al. "What is a medical decision? A taxonomy based on physician statements in hospital encounters: a qualitative study". In: *BMJ Open* 6.2 (2016), e010098. DOI: 10.1136/bmjopen-2015-010098.
